# Supplementary material for: Investigating the neuronal role of the proteasomal ATPase subunit gene PSMC5 in neurodevelopmental proteasomopathies
Source: Nat Commun. 2025 Nov 26;16:10545. doi: 10.1038/s41467-025-65556-8 (PMC12658096; doi:10.1038/s41467-025-65556-8)
Supplement: Supplementary file 1 — Supplementary Information [file 41467_2025_65556_MOESM1_ESM.pdf]

Investigating the neuronal role of the proteasomal ATPase subunit gene *PSMC5* in neurodevelopmental proteasomopathies

Sébastien Küry[1,2,*], Janelle E. Stanton[3,4,§], Geeske M. van Woerden[5,6,7,§], Amélie Bosc-Rosati[8,9,§], Tzung-Chien Hsieh[10,§], Lise Bray[2,§], Marielle Oloudé[11,§], Cory Rosenfelt[12,§], Marie Pier Scott-Boyer[13], Victoria Most[14], Tianyun Wang[15,16,17], Jonas Johannes Papendorf[18], Charlotte de Konink[5,6], Wallid Deb[1,2], Virginie Vignard[2], Maja Studencka-Turski[18], Thomas Besnard[1,2], Anna Marta Hajdukowicz[18], Franziska Thiel[18], Sophie Möller[18], Laëtitia Florenceau[2], Silvestre Cuinat[1,2], Sylvain Marsac[1], Yann Verrès[2], Audrey Dangoumau[19], Léa Poirier[2], Ingrid M. Wentzensen[20], Annabelle Tuttle[20], Cara Forster[21], Johanna Striesow[22], Richard Golnik[23], Damara Ortiz[24], Laura Jenkins[24], Jill A. Rosenfeld[25,26], Alban Ziegler[27], Clara Houdayer[28], Dominique Bonneau[28,29], Erin Torti[20], Amber Begtrup[20], Kristin G. Monaghan[20], Sureni V. Mullegama[20], Catharina M.L. (Nienke) Volker-Touw[30], Koen L.I. van Gassen[30], Renske Oegema[30], Mirjam S. de Pagter[30], Katharina Steindl[31], Anita Rauch[31,32,33,34], Ivan Ivanovski[31], Kimberly McDonald[35], Emily Boothe[36], Andrew Dauber[37], Janice Baker[38], Noelle Andrea V Fabie[38], Raphael A. Bernier[39], Tychele N. Turner[40], Siddharth Srivastava[41], Kira A. Dies[41], Lindsay Swanson[41], Carrie Costin[42], Alali Abdulrazak[43], Rebekah K. Jobling[44], John Pappas[45,46], Rachel Rabin[45], Dmitriy Niyazov[47], Anne Chun-Hui Tsai[48], Karen Kovak[49], David B. Beck[50,51], MCV Malicdan[52,53], David R. Adams[53], Lynne Wolfe[53], Rebecca D. Ganetzky[54,55], Colleen Muraresku[54], Davit Babikyan[56,57], Zdeněk Sedláček[58], Miroslava Hančárová[58], Andrew T. Timberlake[59], Hind Al Saif[60,61], Berkley Nestler[60], Kayla King[60], MJ Hajianpour[62], Gregory Costain[44,63,64], D'Arcy Prendergast[44,65], Chumei Li[66], David Geneviève[67], Antonio Vitobello[68,69], Arthur Sorlin[68,69,70], Christophe Philippe[68,69], Tamar Harel[71,72], Ori Toker[73], Ataf Sabir[74,75], Derek Lim[74,75], Mark Hamilton[76], Lisa Bryson[76], Elaine Cleary[77,78,79], Sacha Weber[80], Trevor L. Hoffman[81], Anna Maria Cueto-González[82,83], Eduardo Fidel Tizzano[82,83], David Gómez-Andrés[84], Marta Codina-Solà[82,83], Athina Ververi[85], Efterpi Pavlidou[86], Alexandros Lambropoulos[87], Kyriakos Garganis[88], Marlène Rio[89], Jonathan Levy[90,91], Sarah Jurgensmeyer[92,93], Anne M. McRae[92], Mathieu K. Lessard[94], Maria Daniela D'Agostino[94], Isabelle De Bie[94], Meret Wegler[95], Rami Abou Jamra[95], Susanne B. Kamphausen[96], Viktoria Bothe[95], Lorraine Potocki[25,97], Eric G. Olinger[98], Yves Sznajer[98], Elsa Wiame[98], Michelle L. Thompson[99], Molly C. Schroeder[99,100], Catherine Gooch[101], Raphael Anthony Smith[102], Arti Pandya[102], Larissa M. Busch[103], Uwe Völker[103], Elke Hammer[103], Kristian Wende[22], Benjamin Cogné[1,2], Bertrand Isidor[1,2], Jens Meiler[14,104], Clémentine Ripoll[105], Stéphanie Bigou[105], Frédéric Laumonnier[19,106], Peter W. Hildebrand[107,108,109], Evan E. Eichler[110,111], Kirsty McWalter[20], Peter M. Krawitz[10], Florence Roux-Dalvai[13], Ype Elgersma[5,6], Julien Marcoux[8,9], Marie-Pierre Bousquet[8,9], Arnaud Droit[112,13], Jeremie Poschmann[11], Andreas M. Grabrucker[113,4,3], Francois V. Bolduc[12,114,115], Stéphane Bézieau[1,2,¥,*], Frédéric Ebstein[2,¥,*], Elke Krüger[18,¥,*]

[1]Nantes Université, CHU Nantes, Service de Génétique Médicale, Nantes, F-44000, France;
[2]Nantes Université, CHU Nantes, CNRS, INSERM, l'institut du thorax, Nantes, F-44000, France;
[3]Bernal Institute, University of Limerick, Limerick, Ireland;
[4]Department of Biological Sciences, University of Limerick, Limerick, Ireland;
[5]Department of Clinical Genetics, Erasmus University Medical Center, Rotterdam, 3015 GD, The Netherlands;

[6]ENCORE Center of Expertise for Neurodevelopmental Disorders, Erasmus Medical Center, Rotterdam, 3015 GD, The Netherlands;
[7]Department of Neuroscience, Erasmus Medical Center, Rotterdam, 3015 GD, The Netherlands;
[8]Institut de Pharmacologie et de Biologie Structurale (IPBS), Université de Toulouse (UT), Toulouse, 31077, France;
[9]Infrastructure Nationale de Protéomique, ProFI, UAR 2048, Toulouse, France;
[10]Institute for Genomic Statistics and Bioinformatics, University Hospital Bonn, Rheinische Friedrich-Wilhelms-Universität Bonn, Bonn, Germany;
[11]Nantes Université, CHU Nantes, INSERM, Center for Research in Transplantation and Translational Immunology, UMR 1064, Nantes, F-44000, France;
[12]Department of Pediatrics, University of Alberta, Edmonton, AB, Canada;
[13]Centre de recherche du CHU de Québec-Université Laval, 2705 boulevard Laurier Québec, Québec, QC, G1V 4G2, Canada;
[14]Institute for Drug Discovery, Medical Faculty, Leipzig University, Leipzig, 4103, Germany;
[15]Department of Medical Genetics, Center for Medical Genetics, Peking University Health Science Center, Beijing, 100191, China;
[16]Neuroscience Research Institute, Peking University; Key Laboratory for Neuroscience, Ministry of Education of China & National Health Commission of China, Beijing, 100191, China;
[17]Autism Research Center, Peking University Health Science Center, Beijing, 100191, China;
[18]Universitätsmedizin Greifswald, Institut für Medizinische Biochemie und Molekularbiologie, Greifswald, 17475, Germany;
[19]Université de Tours, INSERM, Imaging Brain & Neuropsychiatry iBraiN U1253, Tours, 37032, France;
[20]GeneDx, LLC, 207 Perry Parkway, Gaithersburg, MD, 20877, USA;
[21]Loyola University Chicago, Chicago, IL, 60660, USA;
[22]Leibniz Institute for Plasma Science and Technology (INP), Greifswald, 17489, Germany;
[23]Department of Computer Science and Interdisciplinary Center for Bioinformatics, Bioinformatics Group, Universität Leipzig, Härtelstraße 16–18, Leipzig, D-04107, Germany;
[24]UPMC Children’s Hospital of Pittsburgh, One Children’s Hospital Drive, 4401 Penn Avenue, Pittsburgh, PA, 15224, USA;
[25]Department of Molecular and Human Genetics, Baylor College of Medicine, Houston, TX, 77030, USA;
[26]Baylor Genetics Laboratory, Houston, TX, 77021, USA;
[27]Department of Medical Genetics, University Hospital of Toulouse, Toulouse, France;
[28]Service de Génétique médicale, CHU Angers, Angers, France;
[29]Mitovasc, UMR CNRS 6015, INSERM U1083, Angers University, Angers, France;
[30]Department of Genetics, University Medical Centre Utrecht, Utrecht University, Utrecht, 3508 AB, The Netherlands;
[31]Institute of Medical Genetics, University of Zürich, Schlieren-Zurich, 8952, Switzerland;
[32]University Children's Hospital Zurich, Zurich, Switzerland;
[33]University of Zurich Research Priority Program ITINERARE: Innovative Therapies in Rare Diseases, Zurich, Switzerland;
[34]University of Zurich Research Priority Program AdaBD: Adaptive Brain Circuits in Development and Learning, Zurich, Switzerland;
[35]Norton Children's Medical Group, University of Louisville School of Medicine, Louisville, KY, 40202, USA;

[36]University of Mississippi Medical Center, 2500 North State Street, Jackson, MS, 39216, P 601.984.1597, F 601.984.1916, USA;
[37]Division of Endocrinology, Children's National Hospital and Department of Pediatrics, The George Washington University School of Medicine and Health Sciences, Washington, DC, 20010, USA;
[38]Department of Medical Genetics and Genomics, Children's Hospitals and Clinics of Minnesota, Minneapolis, MN, 55404, USA;
[39]Department of Psychiatry & Behavioral Sciences, Center on Human Development and Disability, University of Washington, Box 357920, Seattle, WA, 98195, USA;
[40]Department of Genetics, Washington University School of Medicine, St. Louis, MO, 63110, USA;
[41]Rosamund Stone Zander Translational Neuroscience Center, Department of Neurology, Boston Children's Hospital, Boston, Massachusetts, USA;
[42]Department of Genetics, Akron Children's Hospital, One Perkins Square, Akron, Ohio, 44308, USA;
[43]Division of Genetics, Department of Pediatrics, West Virginia University School of Medicine, One Medical Center Drive, Morgantown, WV, 26506, USA;
[44]Division of Clinical and Metabolic Genetics, The Hospital for Sick Children, Toronto, ON, Canada;
[45]Clinical Genetic Services, Department of Pediatrics, NYU School of Medicine, New York, NY, 10016, USA;
[46]Clinical Genetics, NYU Orthopedic Hospital, New York, NY, 10010, USA;
[47]Division of Medical Genetics, Department of Pediatrics, Duke University School of Medicine, Durham, NC, 22710, USA;
[48]Department of Pediatrics, College of Medicine, University of Illinois, Chicago, IL, 60612, USA;
[49]Department of Molecular and Medical Genetics, Oregon Health and Sciences University, OHSU, Portland, Oregon, USA;
[50]Division of Rheumatology, Department of Medicine, New York University Grossman School of Medicine, New York, NY, USA;
[51]Center for Human Genetics and Genomics, New York University Grossman School of Medicine, New York, NY, USA;
[52]Medical Genetics Branch, National Human Genome Research Institute, NIH, Bethesda, MD, 20892-1851, USA;
[53]National Institutes of Health Undiagnosed Diseases Program, National Human Genome Research Institute, NIH, Bethesda, MD, 20892, USA;
[54]Mitochondrial Medicine Program, Division of Human Genetics & Center for Computational Genomic Medicine, Children's Hospital of Philadelphia, Philadelphia, PA, USA;
[55]Department of Pediatrics, University of Pennsylvania Perelman School of Medicine, Philadelphia, PA, USA;
[56]Department of Medical Genetics, Yerevan State Medical University after Mkhitar Heratsi, Yerevan, Armenia;
[57]Laboratory of Molecular Genetic, Center of Medical Genetics and Primary Health Care, Yerevan, Armenia;
[58]Department of Biology and Medical Genetics, Charles University 2nd Faculty of Medicine and University Hospital Motol, Prague, 150 06, Czech Republic;
[59]Wyss Department of Plastic Surgery, NYU Langone Medical Center, New York, NY, USA;
[60]Department of Human and Molecular Genetics, Division of Clinical Genetics, Virginia Commonwealth University School of Medicine, Richmond, VA, USA;

[61]Department of Pediatrics, Division of Clinical Genetics, Virginia Commonwealth University School of Medicine, Richmond, VA, USA;
[62]Division of Medical Genetics and Genomics, Department of Pediatrics, Albany Medical College, Albany, NY, USA;
[63]Department of Molecular Genetics, University of Toronto, Toronto, ON, M5S 1A4, Canada;
[64]Program in Genetics and Genome Biology, SickKids Research Institute, Toronto, ON, Canada;
[65]Department of Paediatrics, Temerty Faculty of Medicine, University of Toronto, Toronto, ON, Canada;
[66]McMaster University Medical Center, Hamilton, ON, Canada;
[67]Université Montpellier, Inserm U 1183, Centre de référence maladies rares anomalies du développement, Service de génétique médicale, Hôpital Arnaud de Villeneuve, Montpellier, France;
[68]UMR 1231 GAD, Inserm, Université de Bourgogne Franche Comté, Dijon, France;
[69]Unité Fonctionnelle Innovation en Diagnostic Génomique des Maladies Rares, Fédération Hospitalo-Universitaire-TRANSLAD, CHU Dijon Bourgogne, Dijon, France;
[70]Centre de Génétique et Centre de Référence Anomalies du Développement et Syndromes Malformatifs de l'interrégion Est et FHU TRANSLAD, Centre Hospitalier Universitaire de Dijon, Dijon, France;
[71]Department of Genetics, Hadassah Medical Organization, Jerusalem, Israel;
[72]Faculty of Medicine, Hebrew University of Jerusalem, Jerusalem, Israel;
[73]Department of Pediatrics, Allergy and Clinical Immunology Unit, Shaare Zedek Medical Center, Faculty of Medicine, Hebrew University of Jerusalem, Jerusalem, Israel;
[74]Clinical Genetics Department, Birmingham Women's and Children's NHS Foundation Trust, Birmingham, United Kingdom;
[75]Institute of Cancer and Genomic Sciences, University of Birmingham, Birmingham, UK;
[76]West of Scotland Clinical Genetics Service, Queen Elizabeth University Hospital, Glasgow, UK;
[77]South East Scotland Genetics Service, Western General Hospital, Edinburgh, UK;
[78]Centre for Clinical Brain Sciences, University of Edinburgh, Edinburgh, UK;
[79]UK Dementia Research Institute at University of Edinburgh, University of Edinburgh, Edinburgh, EH16 4SB, UK;
[80]Service de Génétique Médicale, Hôpital Armand-Trousseau, APHP, Sorbonne Université, Paris, France;
[81]Department of Genetics, Southern California Kaiser Permanente Medical Group, Anaheim, CA, 92801, USA;
[82]Department of Clinical and Molecular Genetics, Vall d'Hebron Hospital Universitari, Vall d'Hebron Barcelona Hospital Campus, Barcelona, Spain;
[83]Medicine Genetics Group, Vall d'Hebron Institut de Recerca (VHIR), Vall d'Hebron Hospital Universitari, Vall d'Hebron Barcelona Hospital Campus, Barcelona, Spain;
[84]Pediatric Neurology, Vall d'Hebron Institut de Recerca (VHIR), Vall d'Hebron Hospital Universitari, Vall d'Hebron Barcelona Hospital Campus, Barcelona, Spain;
[85]Department of Genetics for Rare Diseases, 'Papageorgiou' General Hospital, Thessaloniki, 56429, Greece;
[86]Department of Speech and Language Therapy, University Hospital of Ioannina, Ioannina, 45111, Greece;
[87]Genetic Unit, 1st Department of Obstetrics and Gynecology, School of Medicine, Aristotle University of Thessaloniki, 'Papageorgiou' General Hospital, Thessaloniki, 56429, Greece;

[88]Epilepsy Unit, St Luke's Hospital, Thessaloniki, Greece;
[89]Service de Médecine Génomique des Maladies Rares, Hôpital Necker-Enfants Malades, AP-HP, Paris, 75015, France;
[90]Department of Genetics, APHP-Robert Debré University Hospital, Paris, 75019, France;
[91]Multi-site medical biology laboratory SeqOIA - FMG2025, Paris, 75014, France;
[92]Division of Genetics, Genomics and Metabolism, Ann & Robert H. Lurie Children's Hospital of Chicago, Chicago, IL, USA;
[93]Department of Pediatrics, Northwestern University Feinberg School of Medicine, Chicago, IL, USA;
[94]Division of Medical Genetics, Department of Specialised Medicine, McGill University Health Centre, Department of Human Genetics, McGill University, Montreal, QC, Canada;
[95]Institute of Human Genetics, University of Leipzig Medical Center, Leipzig, 04103, Germany;
[96]Institute of Human Genetics, University Hospital Magdeburg, University Hospital Magdeburg, Magdeburg, 39120, Germany;
[97]Texas Children's Hospital, Houston, TX, USA;
[98]Center for Human Genetics, Cliniques Universitaires Saint-Luc, UCLouvain, Avenue Hippocrate 10, Brussels, 1200, Belgium;
[99]Department of Pathology and Immunology, Division of Laboratory and Genomic Medicine, Washington University School of Medicine in Saint Louis, St. Louis, MO, USA;
[100]Department of Pediatrics, Washington University School of Medicine, St. Louis, MO, USA;
[101]Department of Pediatrics, Division of Genetics and Genomic Medicine, Washington University in St Louis, St. Louis, MO, USA;
[102]Department of Pediatrics, Division of Genetics and Metabolism, University of North Carolina Health, Chapel Hill, NC, USA;
[103]Universitätsmedizin Greifswald, Interfakultäres Institut für Genetik und Funktionelle Genomforschung, Abteilung für Funktionelle Genomforschung, Greifswald, 17475, Germany;
[104]Center for Structural Biology, Vanderbilt University, Nashville, TN, 37240, USA;
[105]ICV-iPS core facility, Sorbonne Université, Institut du Cerveau - Paris Brain Institute - ICM, Inserm, CNRS, APHP, Hôpital de la Pitié Salpêtrière, Paris, France;
[106]Service de Génétique, Centre Hospitalier Régional Universitaire, Tours, 37044, France;
[107]Institut für Medizinische Physik und Biophysik, Universität Leipzig, Medizinische Fakultät, Härtelstr. 16-18, Leipzig, 04107, Germany;
[108]Charité Universitätsmedizin Berlin, Corporate member of Freie Universität Berlin and Humboldt-Universität zu Berlin, Institute of Medical Physics and Biophysics, Berlin, Germany;
[109]Berlin Institute of Health, Berlin, 10178, Germany;
[110]Department of Genome Sciences, University of Washington School of Medicine, Seattle, WA, 98195, USA;
[111]Howard Hughes Medical Institute, University of Washington, Seattle, WA, 98195, USA;
[112]Département de médecine moléculaire, Faculté de médecine, Université Laval, 2325 rue de l'Université, Québec, QC, G1V 0A6, Canada;
[113]Health Research Institute (HRI), University of Limerick, Limerick, Ireland;
[114]Neuroscience and Mental Health Institute, University of Alberta, Edmonton, AB, Canada;
[115]Department of Medical Genetics, University of Alberta, Edmonton, AB, Canada;
[§]These authors contributed equally;
[¥]These authors supervised jointly;
***Corresponding authors: E-mails: sebastien.kury@chu-nantes.fr (S.K.); stephane.bezieau@chu-nantes.fr (S.B.); frederic.ebstein@univ-nantes.fr (F.E.) ; elke.krueger@uni-greifswald.de (E.K.)

Supplementary information

Supplementary methods

Plasmid construction and transfection

The cDNA for *PSMC5* coding wild-type PSMC5 (i.e. RPT6) full-length protein was amplified by RT-PCR from total RNA isolated from HeLa cells and cloned into the pcDNA3.1/Zeo(+) (Invitrogen) using the *Kpn* I and *Xho* I restriction sites. For N-terminal tagging, oligonucleotides encoding a tandem HA epitope (GYPYDVPDYAMGGYPYDVPYAGT) were constructed, annealed and inserted in frame into the pcDNA3.1/Zeo(+) expression vector containing *PSMC5* to generate a HA-PSMC5 (i.e. HA-Rpt6) fusion protein. Thirteen cDNA variant constructs were then prepared according to mRNA sequence NM_002805.6, and contained respectively the variants c.414G>C p.(Pro183Leu); c.601C>T p.(Arg201Trp); c.605C>T p.(Ala202Val); c.620C>T p.(Thr207Met); c.647G>A p.(Gly216Asp); c.662A>G p.(Gln221Arg); c.749A>T p.(Glu250Val); c.772C>T p.(Arg258Trp); c.959C>G p.(Pro320Arg); c.959C>A p.(Pro320His); c.973C>T p.(Arg325Trp); c.1103T>C p.(Met368Thr); c.1182_1183del p.(Asp394Glufs*2). These *PSMC5* variants were introduced into the pcDNA3.1/HA-*PSMC5* plasmid by site-directed mutagenesis, using appropriate primer pairs designed with the Quick-Change Primer Design software from Agilent. The JetPRIME reagent (Polyplus-transfection® SA) was employed to transfect SH-SY5Y cells according to the manufacturer's instructions.

Transcriptomic analyses on human T cells

Bioinformatics: primary analysis. The BCL files generated by the sequencer were demultiplexed using bcl2fastq2 ("Bcl2fastq2 Conversion Software v2.20 Software Guide (15051736)"), producing FASTQ files containing a total of 342.3 million paired reads. The resulting FASTQ files were analyzed using the 3' Sequencing RNA Profiling (SRP) pipeline [1]. This methodology aligns with previous DGEseq analyses performed in various studies [2, 3, 4]. The SRP pipeline integrates cutadapt v4.5 [5] for read trimming and bwa aln v0.7.17 [6] for

alignment to the hg38 human reference genome and transcriptome. A custom Python script integrated into the SRP pipeline was used to parse and count UMIs, generating a raw matrix containing transcript counts, with columns representing samples and rows representing genes. The dataset included a total of 28,307 genes and 32 samples, as sequencing was performed in technical replicates.

First series of proteomic analyses on human T cells

To decode the cellular effects of *PSMC5* loss-of-function causing NDD, the proteomes of T cells from subjects with PSMC5 variants were analyzed in two independent series. In the first series, samples from subjects S7 and S14 were analyzed using mass spectrometry-based proteomics and compared to those of unaffected parents (mother and father, respectively). In order to determine if the results could be replicated, a second series or proteomic analyses was performed on samples from subjects S14 (p.Gln221Arg), S15 (p.Glu250Val), S25 (p.Pro320Arg), and samples from S36 (p.Arg325Trp) and S37 (p.Arg325Trp) pooled together in a single sample; proteomes of affected subjects were compared to those of seven unrelated unaffected individuals (see the main section of the materials and methods for a detailed description of the methodology). The methods described below refers to the first series of analyses.

Preparation of protein samples. Protein was extracted from primary T cells by five cycles of freezing (liquid nitrogen) and thawing (30°C, 1,400 rpm) in 8 M urea/ 2 M thiourea. Cell debris and insoluble material was separated by centrifugation (16,000 × g, 1 h at 20 C). Protein content was determined with a Bradford assay (Biorad, Munich, Germany).

Sample preparation for mass spectrometry. Four µg of total protein from each sample were reduced (2.5 mM DTT ultrapure, Invitrogen, for 15 min at 37 °C) and alkylated (10 mM iodacetamide, Sigma Aldrich, for 30 min at 37°C). Benzonase (Novagen, Merck Millipore, Darmstadt Germany) 0.6 U/µg protein) was applied for nucleic acid degradation before digestion with trypsin (Promega, Madison, WI, USA) at an enzyme to protein ratio 1:25 over night at 37 °C. The tryptic digestion was stopped by adding acetic acid (final concentration 1%)

followed by desalting using ZipTip-µC18 tips (Merck Millipore, Darmstadt, Germany). Eluted peptides were concentrated by evaporation under vacuum and subsequently resolved in 0.1% acetic acid, 2% acetonitrile (ACN) containing HRM/iRT peptides (Biognosys, Zurich, Switzerland) according to manufacturer's recommendation. For the generation of a spectral library a protein pool of equal protein amounts (10 µg) of each sample was prepared as described above. Resulting peptides were desalted (SepPak, Waters, Eschborn, Germany) and fractionated by strong cation exchange chromatography (PolySulfoethyl A™ column, 150 x 1mm, 5µm, 200A, Poly LC Inc., Columbia, ML, USA). Peptide fractions (n=17) were purified for mass spectrometric analyses as described above.

Mass Spectrometry Measurements. Mass spectrometric (MS) data was recorded on a QExactive HF mass spectrometer (Thermo Electron, Bremen, Germany). Before MS data acquisition tryptic peptides were separated by reverse phase chromatography (Accucore 150-C18, 25 cm x 75 µm, 2,6 µm C18, 150 Å) using an Ultimate 3000 nano-LC system (both Thermo Scientific, Waltham, MA, USA) at a constant temperature of 40°C and a flow rate of 300 nL/min. To design a spectral library, MS/MS peptides were separated by 120 min linear gradients with increasing acetonitrile concentration from 5 to 25 % in 0.1 % acetic acid. Data were recorded in data dependent mode (DDA). The acquisition of MS data for relative quantitation was performed in data independent mode (DIA) after peptide pre-fractionation at chromatographic conditions described above. For further details to the instrumental setup and the parameters for LC-MS/MS analysis in DDA and DIA mode, see Supplementary Table 3a,b.

Data analysis. Proteins were identified using Spectronaut™ Pulsar 13.9 software (Biognosys AG) against a spectral library generated from data-dependent acquisition measurements of a SCX-fractionated peptide pool. The spectral library construction by Spectronaut was based on a database search using a human protein database (Uniprot vs 03_2019, 20404 entries) (Supplementary Table 3c). The generation of the ion library in Spectronaut™ v13.9.191106.43655 resulted in a constructed library consisting of 920,617 fragments, 67,323 peptides and 6717 protein groups. The Spectronaut DIA-MS analysis was carried out as described previously[7] with project specific modifications (Supplementary Table

3c). Peptides were assigned to protein groups and protein inference was resolved by the automatic workflow implemented in Spectronaut. Only proteins with at least two identified peptides were considered for further analyses. Data analysis was performed with an in-house R-pipeline. Data was median normalized on ion level. Statistical analysis was carried out on peptide level using the algorithm ROPECA[8]. Peptides with oxidized methionine were not included in the quantitative analysis. Binary differences have been identified by application of a moderate paired t-test[9]. Family was used as pairing variable. Multiple test correction was performed according to Benjamini-Hochberg. Variance within the data set was visualized by principal component analyses and differences in the protein pattern by Volcano plots (data not shown); for representation of protein intensities Hi3 Peptides were used. Data obtained for the quantification of Hi3 peptides are reported in Supplementary Table 3d,e. Supplementary Table 3d reports the separate comparisons between the individual intensities of Hi3 peptides obtained for Subject S6 and her mother, on the one hand, and Subject S14 and her father, on the other hand. Supplementary Table 3e reports the comparisons between combined intensities of Hi3 peptides from the affected individuals (Mut= Subjects S7 and S14) and combined intensities from their parental controls (CO= Subject S6's mother and Subject S14's father).

The mass spectrometry proteomics data have been deposited to the ProteomeXchange Consortium via the PRIDE[10] partner repository with the dataset identifier PXD048558.

Functional enrichment analysis from proteomics data. The statistical analysis was performed on proteins with more than two peptides, using intensity data obtained by Hi3 peptides quantification reported in Supplementary Table 3e. All proteins with p-value<0.05 were included in the functional enrichment (Supplementary Fig. 6a). The functional analysis was performed using the R package gprofiler2 was used to perform pathway enrichment analyzes (DB: GO:BP,KEGG, REAC and WP) with gene symbols as input. The following options were selected: correction_method=fdr, user_threshold=0.05.

A Fisher's exact test was performed to determine if the significant proteins were enriched for specific biological terms of interest: The input data consisted of a list of proteins that were differentially expressed between patients and controls (p-value < 0.05). (i) The reference database containing information about mitochondrial genes was the MitoCarta3.0 human inventory implemented by the Broad Institute (https://www.broadinstitute.org/files/shared/metabolism/mitocarta/human.mitocarta3.0.html); it consists in ‘a collection of 1136 nuclear and mtDNA genes encoding proteins with strong support of mitochondrial localization’[11]. (ii) The aging gene list was obtained by searching the query ('longevity' OR 'lifespan' OR 'CLS' OR 'chronological life span' OR 'ageing') AND 'Homo sapiens'[porgn:__txid9606] on https://www.ncbi.nlm.nih.gov/gene (Supplementary Table 4a). The enrichment test assessed whether certain biological terms were overrepresented in the input list compared to what would be expected by chance, using a Fisher's exact test.

Second series of proteomic analyses on human T cells

Functional enrichment analysis from proteomics data. The proteins were classified according to gene lists corresponding to different pathways; Neuroinflammatory responses (GO:0150076, GO:0150077, GO:0150078, GO:0150079), cell adhesion (GO:0007155), learning and memory (GO:0150076), lamin and apolipoproteins, determination of adult lifespan (GO:0008340 and an exhaustive list of genes associated with aging reported in Supplementary Table 4a) and Alzheimer markers (list established by Pedrero-Prieto *et al*. [12] and reported in Supplementary Table 4b). Those different groups are colored as visualized on the volcano plot. Cytoscape software version 3.10.1 was used to visualize the functional enrichment and the different pathways dysregulated in patients compared to healthy controls. The significant proteins with the threshold of *p*-value < 0.05 and the -2 ≤ Fold Change ≤ 2 were selected and clustered according to Gene Ontology (GO), Kyoto Encyclopedia of Genes and Genomes (KEGG), and Search Tool for the Retrieval of Interacting Genes/proteins (STRING). The clustering was handled by the Markov Clustering algorithm (MCL) with a granularity parameter set at 4 [13].

Supplementary results

Three-dimensional (3D) structural analysis predicts that missense *PSMC5* variants affect proteasome function in various ways

Most *PSMC5* variants affect residues located near neighboring ATPases from the base of the 19S regulatory particle. For instance, Met368 appears to interact with PSMC4/Rpt3 by forming interactions with the hydrophobic moieties of Tyr191, Pro199 and Arg 329 (Supplementary Fig. 2d). Its substitution by a polar threonine, thereby creating a change in the electrostatic environment on the subunit's interface, might compromise complex formation and the integrity of this part of the 26S proteasome. Furthermore, 6/13 residues play a role in the inter- and intramolecular rearrangements that occur during the transition from substrate-free to substrate-engaged states of the 26S proteasome (Supplementary Fig. 2b), suggesting that alteration of these amino acids could disrupt this phase. For example, Arg201 in PSMC5/Rpt6 forms a polar interaction network with PSMC4/Rpt3 near the ATP-binding pocket (Supplementary Fig. 2b). Glu250 is close to the ATP binding site though not close enough to form a contact with the charged phosphate (Supplementary Fig. 2b). Since ATP binding appears to be essential for proteasome assembly[14], modifications of amino acids at the ATP binding pocket may affect ATP binding and hydrolyzation and thereby the integrity of the tertiary structure. This would explain the drastic effect of these two variants on *PSMC5* expression. Arg258 forms a contact network with PSMC1/Rpt2 near the substrate binding channel (Fig. 1b and Supplementary Fig. 2c). As this variant is well expressed and incorporated in *in vitro* experiments, it stands to reason that it mainly plays a role in substrate processing, although we did not further confirm its effect on proteasome activity in our analysis. In presence of this variant, the chemical identity of the side chain is altered, which could influence the dynamics of the subunit and thus substrate processing. In addition to a change in the physicochemical properties of the side chains, there are some variants that are more likely to affect the flexibility or rigidity of the protein backbone. We identified four variants in which either a proline or a glycine is altered (Pro183Leu, Gly216Asp, Pro320Arg, Pro320His). Glycine typically allows for backbone flexibility, while proline enforces conformational rigidity

and often induces turns. Predicting the exact effects of these four variants on protein folding and activity remains challenging. However, since both Pro183 and Pro320 are situated within turns between two secondary structure elements (Fig. 1B and Supplementary Fig. 3), it is highly likely that these changes will impact protein folding and incorporation of subunits. Furthermore, Pro320 and Arg325 (Arg325Trp) are part of the P-loop between the core and the AAA+-lid domain. Arg325 forms multiple hydrogen bonds connecting these two domains (Supplementary Fig. 3). Altogether, these findings strongly suggest that substitutions in PSMC5/Rpt6 are likely to have multifaceted effects on proteasome.

PSMC5 loss-of function is associated with proteomic changes affecting immunity, metabolism and cell proliferation

Pairwise comparison and hierarchical clustering analysis of proteomics data identified approximately 20 proteins significantly enriched in subjects S6 and S11 compared to their parents (Supplementary Fig. 6a): (i) players of the innate and adaptive immune responses, such as APOBEC3G or HLA class I (HLA-A) and II (HLA-DRB1) molecules, which are typically induced by type I and II IFN, respectively[15]; (ii) a set of apolipoproteins (APOA1, APOE, APOA2, APOC1, APOC3, APOA4, APOBR and APOL3) (iii) proteasome subunit PSMB7 (i.e. β2), suggesting that T cells with *PSMC5* variants attempt to restore protein homeostasis by inducing the synthesis of new proteasome complexes, as previously described[16]; (iv) prelamin A (LMNA); (v) proteins related to innate immunity (PPP1R11), (vi) amino acids (FAH), or (vii) glutathione (GSTM1). Note that some of these proteins were also relatively abundant in S10's father –exhibiting moderate NDD- who transmitted alteration *PSMD11* NM_002815.3:c.268C>T p.(Arg90*) to his daughter, confirming the likely pathogenicity of this second proteasome variant. Finally, a group of proteins were consistently downregulated across NDD patients, most of them regulators of cell proliferation, such as CDC123, REPS1 and AAGAB (Supplementary Fig. 6a). Altogether, these investigations uncovered a series of candidate biomarkers for NDD caused by proteasome defects which themselves reflect profound remodeling of immune responses and lipid metabolism.

A Fisher's exact test was performed to determine if the significant proteins were enriched for specific biological terms of related either to mitochondria or aging. According to the lists provided for both biological terms, amongst all the proteins that could be tested by proteomic analysis, 4 of the 55 proteins found significantly deregulated (p-value<0.05) are linked to mitochondria (NIT1, MTHFD2, GPX4, TRIAP1 on volcano plot in Supplementary Fig. 6b). However, the p-value of hypergeometric test related to the whole list of mitochondrial genes is not significant (p-value = 0.9). By comparison, 6 of the 55 significantly deregulated proteins are related (LMNA, APOE, HLA-DRB1, HLA-DQA1, APOC3, GSTM1 on volcano plot in Supplementary Fig. 6c), with a significant p value of the hypergeometric test related to the list of aging genes (p-value = 0.007).

Supplementary figures and tables

Supplementary Table 1: Predictions and functional tests for the PSMC5 variants identified in the affected individuals included in the study.

Supplementary Table 2: Clinical features of the subjects with *PSMC5* variants and indels.

Supplementary Table 2a: Detailed clinical features of the subjects with *PSMC5* variants and indels.

Supplementary Table 2b: Clinical features described by Human Phenotype Ontology (HPO) terms across the patient cohort.

Supplementary Table 2c: Individual lists of symptoms and signs observed in each affected subjects of the study.

Supplementary Table 3: First series of proteomics analyses.

Supplementary Table 3a: LC-MS/MS parameter (data dependent mode, spectral library).

Supplementary Table 3b: LC-MS/MS parameter (data independent mode; quantitative data).

Supplementary Table 3c: Peptide and protein identification parameters in Spectronaut.

Supplementary Table 3d: Results of proteome-wide analysis indicated by Hi3 peptide intensity data: comparison at the family level between individual data from affected individuals S6 and S11 and individual data from their parental controls.

Supplementary Table 3e: Results of proteome-wide analysis indicated by Hi3 peptide intensity data: comparison between combined data from affected individuals S6 and S11 and combined data from their parental controls.

Supplementary Table 4: Datasets of genes used for functional enrichment analysis from proteomics and transcriptomics data.

Supplementary Table 4a: List of genes related to aging used for functional enrichment analysis from proteomics and transcriptomics data.

Supplementary Table 4b: List of genes suggested as biomarkers of Alzheimer's disease in cerebrospinal fluid by Pedredo-Prieto *et al.* used for functional enrichment analysis from proteomics and transcriptomics data.

Supplementary Table 5: Materials used in this study.

Supplementary Table 5a: Reagents, software, and equipment used in this study.

Supplementary Table 5b: Oligonucleotide sequences used in this study.

Supplementary Table 5c. Animal strains used in this study.

Supplementary Table 5d. Cell lines used in this study.

Supplementary Table 5e. Web resources for prediction of variant pathogenicity and 3D modeling.

Supplementary Table 6. List of participating teams and ethics committees.

Supplementary Table 6a. List of diagnostic or research teams involved in this study.

Supplementary Table 6b. List of local ethics committees that approved subject inclusion and sample collection.

Supplementary Fig. 1: Variants in sub-unit proteasome genes can induce two distinct phenotypes.

Supplementary Fig. 2: Evolutionary conservation of the PSMC5/Rpt6 residues subjected to variations associated with NDD.

Supplementary Fig. 3: Structural analysis of *PSMC5* variants shows that most missense variants are located close to neighboring ATPases PSMC1/Rpt2 and PSMC4/Rpt3.

Supplementary Fig. 4: The localization of recurrent variants p.Pro320Arg and p.Arg325Trp in the 19S particle provide clues to the 19S-20S assembly defects induced.

Supplementary Fig. 5: Distribution of mean pairwise distance between facial features of subjects with *PSMC5* variants.

Supplementary Fig. 6: *PSMC5* variants differentially affect protein turnover and proteasome assembly in SHSY5Y cells.

Supplementary Fig. 7: Proteome profiling of T cells from NDD subjects identifies candidate biomarkers for proteasome dysfunction.

Supplementary Fig. 8: Transcriptomics of T cells from NDD subjects with *PSMC5* heterozygous variants confirms type I IFN response and suggests possible impairment of ribosome biogenesis.

Supplementary Fig. 9: T cells from NDD subjects with *PSMC5* heterozygous variants exhibit a strong upregulation of ISG.

Supplementary Fig. 10: Sterile type I interferon (IFN) responses triggered in NDD subjects with *PSMC5* heterozygous variants predominantly rely on the ISR.

Supplementary Fig. 11: Transcriptomics of ectodermal cells derived from PSMC5$^{(+/p.Arg325Trp)}$ iPSCs highlights altered sterol and lipid metabolism and impairment of the process related to the development of craniofacial and conotroncal structures.

Supplementary Fig. 12: Transcriptomics of mesodermal cells derived from PSMC5$^{(+/p.Arg325Trp)}$ iPSCs highlights increased viral/interferon response and altered developmental processes.

Supplementary Fig. 13: Example of the gating strategy used for analyses by flow cytometry.

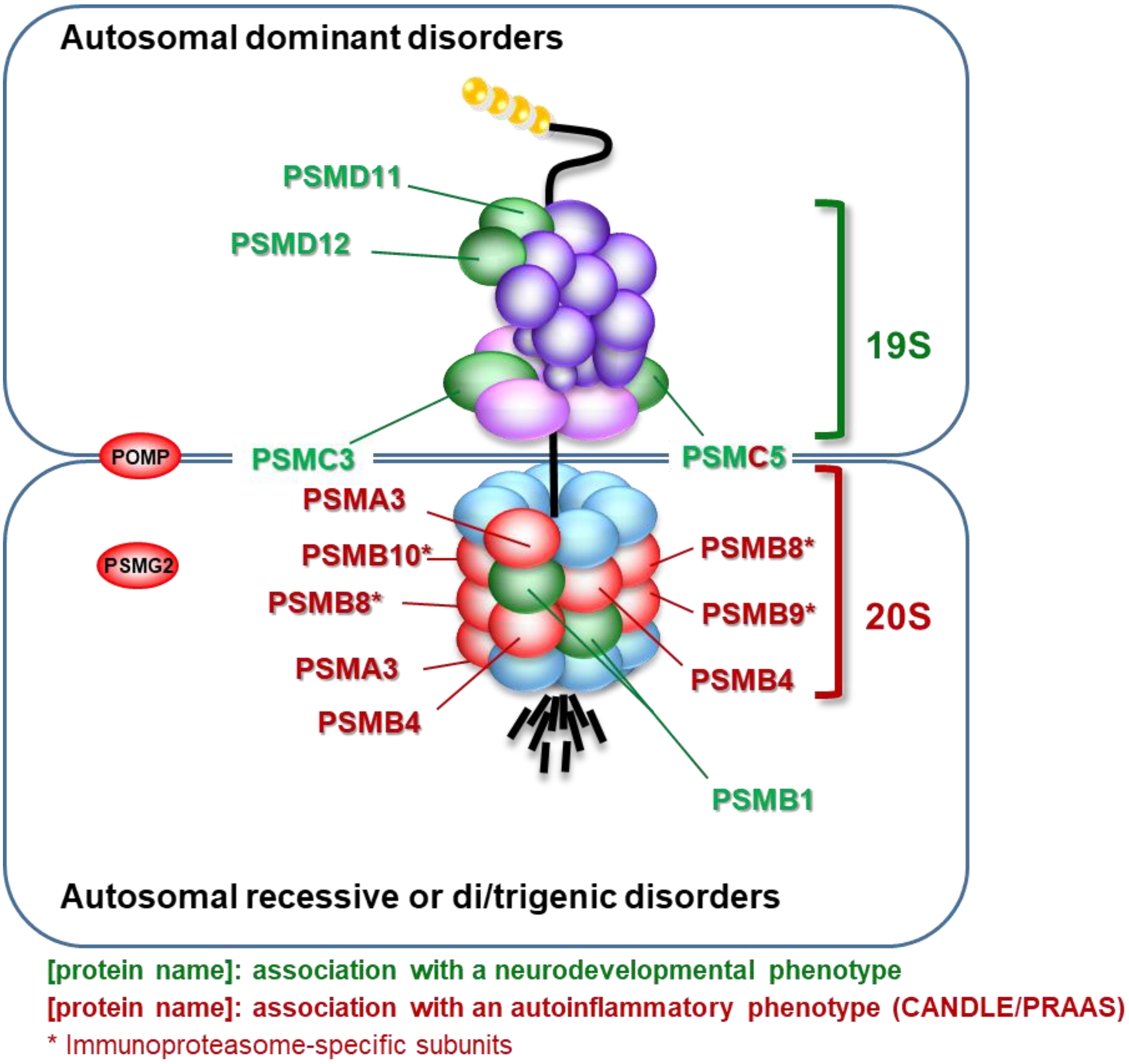

Supplementary Fig. 1: Variants in sub-unit proteasome genes can induce two distinct phenotypes. In this schematic view of a 26S proteasome, subunits colored in green correspond to those associated with a neurodevelopmental phenotype, while those colored in red are associated with an autoinflammatory phenotype. Assembly helpers are represented by red ovals. Typically, NDDs are autosomal dominant disorders caused by heterozygous variants, usually novo, of 19S regulatory particle genes [17, 18, 19, 20]. In contrast, CANDLE/PRAAS-type autoinflammatory diseases are either autosomal recessive disorders caused by bi-allelic (homozygous or compound heterozygous) variants in 20S particle genes, or disorders resulting from a digenic mechanism involving heterozygous variants in two distinct genes of the 20S particle[21]. However, this dichotomy is very relative, as a number of exceptions have been observed. We can cite, for example, a NDD caused by bi-allelic variants of *PSMB1*[22], a gene coding a 20S subunit, a familial form of NDD caused by a homozygous variant in *PSMC3*[23], autosomal dominant PRAAS cases caused by heterozygous variants of PSMB9 or PSMB8, or a CANDLE/PRAAS case resulting from a potential trigenic mechanism involving heterozygous variants in *PSMB8*, *PSMA5* and *PSMC5*[24]-hence the two-tone green and red lettering used on the figure for PSMC5.

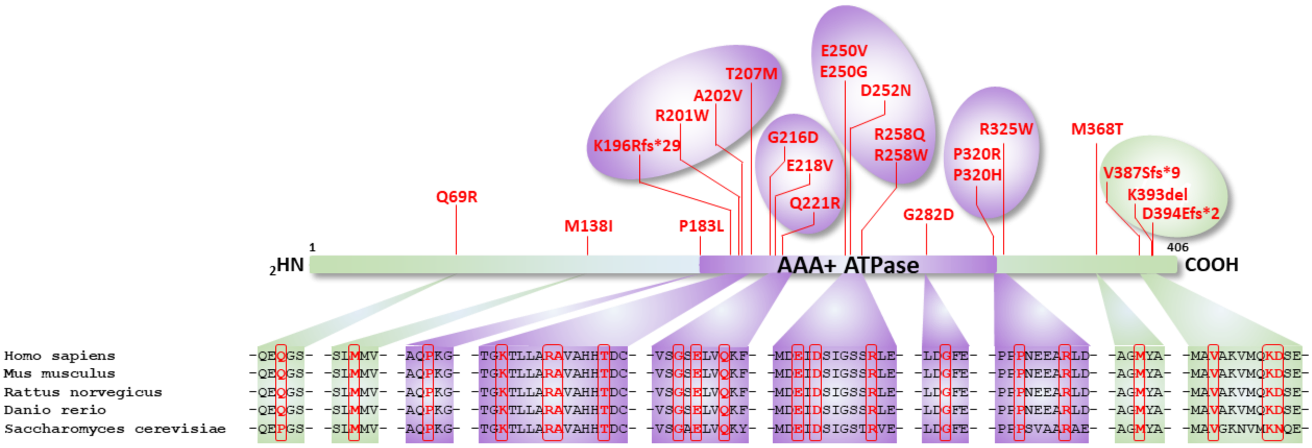

Supplementary Fig. 2: Evolutionary conservation of the PSMC5/Rpt6 residues subjected to variations associated with NDD. Alignment of the primary structures of human, mouse, rat, zebrafish and yeast PSMC5/RPT6 showing that all affected residues are evolutionary well conserved across species. Purple clouds indicate clusters of variants within or in proximity to the AAA+ ATAase domain, whereas the green cloud indicate a cluster of variants observed outside this domain.

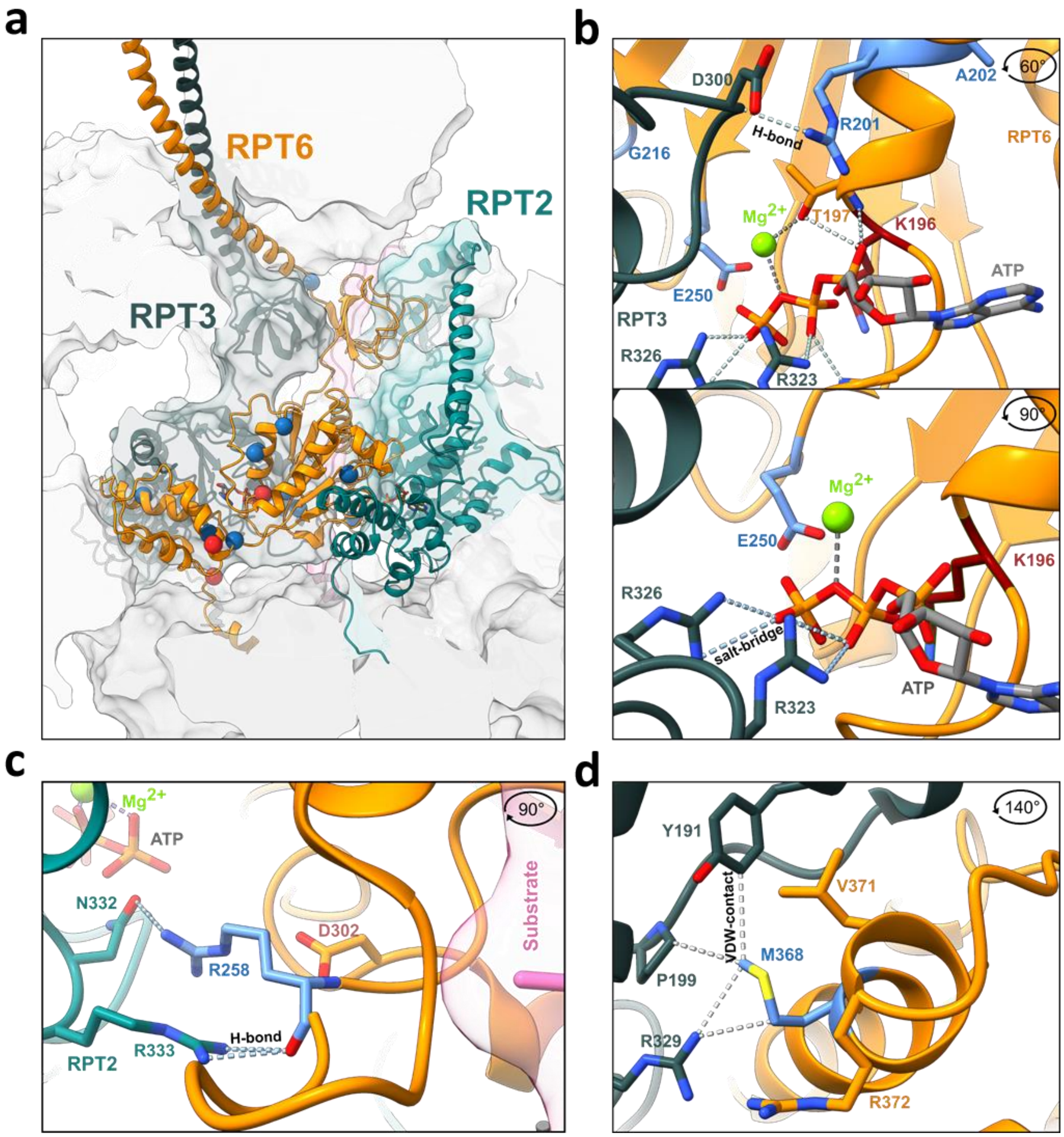

Supplementary Fig. 3: Structural analysis of *PSMC5* variants shows that most missense variants are located close to neighboring ATPases PSMC1/Rpt2 and PSMC4/Rpt3. a General overview. The variants or interest (blue spheres for missense variants and red spheres for frameshift variants) are visualized within the structure of Rpt6/PSMC5 (orange) in the substrate engaged Cryo-EM structure 6MSK and the substrate free structure 7W37[25, 26]. They are primarily located in the lower ATPase domain close to interfaces to the Rpt2/PSMC1 (dark green), Rpt3/PSMC4 (light green), and the substrate (pink). **b-d** In a significant number of the variants identified in affected individuals, subunit interfaces are modified, as illustrated by the (**b**) detailed view of Glu250 (E250; p.Glu250Gly, p.Glu250Val), Arg201 (R201; p.Arg201TRp) and Lys196 (K196; p.Lys196Argfs*29) which forms bonds with the residues Arg323 (R323) and Arg326 (R326) of Rpt3/PSMC4, (**c**) detailed view of Arg258 (R258, p.Arg258Trp, p.Arg258Gln) which forms hydrogen bonds with Asp300 (D300), Arg233 (R233) and Asn332 (N332) of Rpt2/PSMC1, respectively, and (**d**) and detailed view of Met368 (M368; p.Met368Thr) which forms hydrophobic interactions with residues in the small subdomain of the Rpt3 ATPase domain. Hydrophobic interactions are indicated as grey dashed lines.

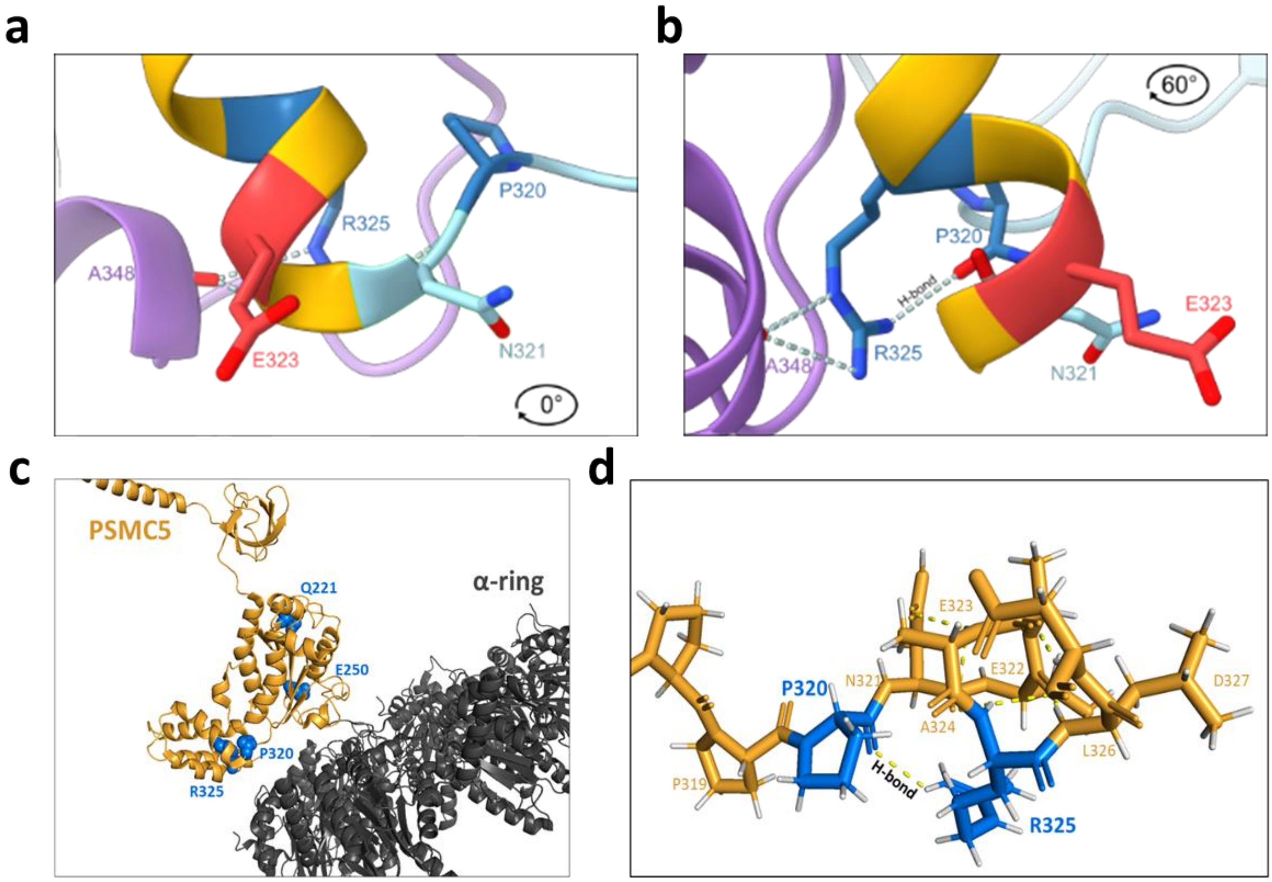

Supplementary Fig. 4: The localization of recurrent variants p.Pro320Arg and p.Arg325Trp in the 19S particle provide clues to the 19S-20S assembly defects induced. a-b. Detailed view of the Rpt6 region subject to recurrent variants V19:Pro320 (P320; p.Pro320Arg, p.Pro320His) and V22:Arg325 (R325; p.Arg325Trp). The residue Glu323 (E323), predicted to be deleted by variant V21: c.970-2A>G (p.Glu323del) is also indicated. **c-d.** Structural analysis suggests that the 19S-20S assembly defect may be due to a structural dysorganization induced by the localization of the variants in the 19S particle. From the general overview of 20S α-ring and PSMC5 inferred from PDB structure 7W37 (**c**), we observe that P320 and R325 (blue spheres) are very close to each other and located on the 10th alpha helix at the 20S α-ring interface (black). The two variants are connected by a strong hydrogen bond (**b,d**), which could help maintain the structure of the alpha helix.

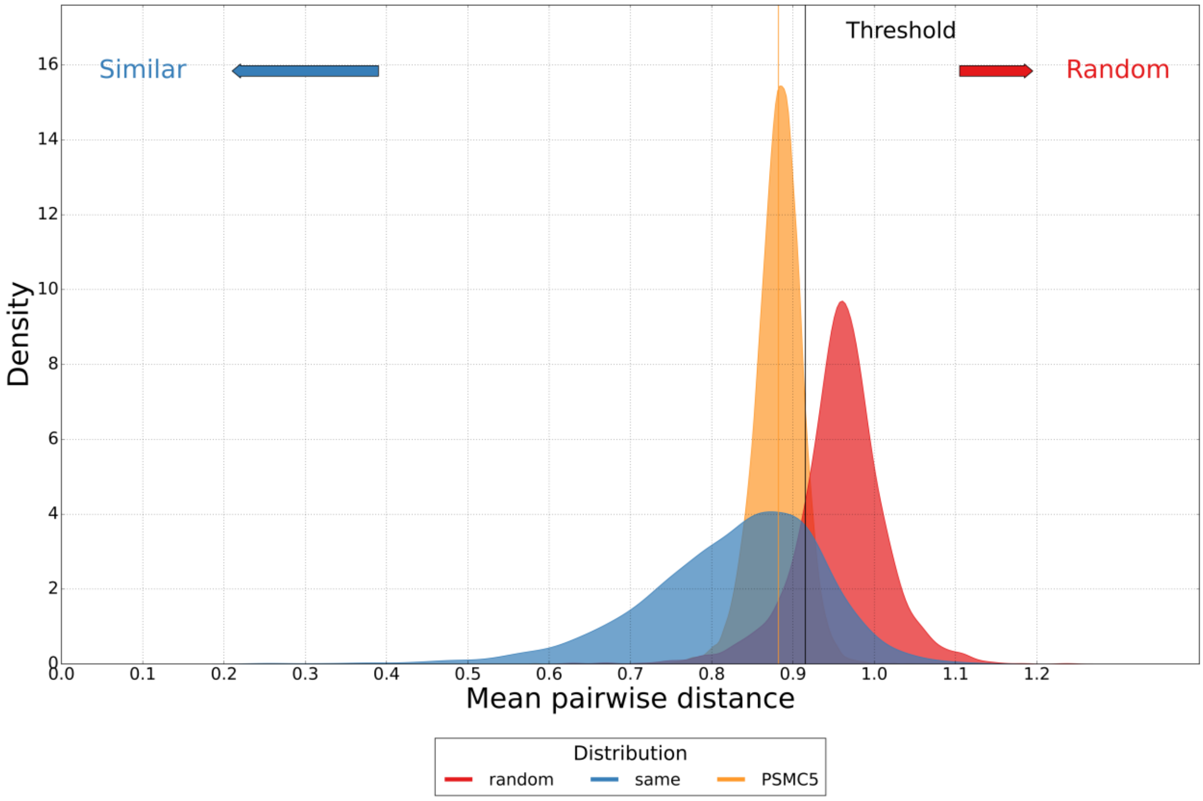

Supplementary Fig. 5: Distribution of mean pairwise distance between facial features of subjects with *PSMC5* variants. It shows three distributions: *PSMC5*, the random selection from the subjects with 328 disorders, and the selection with the same disorder. Black vertical line is the threshold that classifies whether it is the same disorder or random selection. 90% of *PSMC5* distribution are below the threshold.

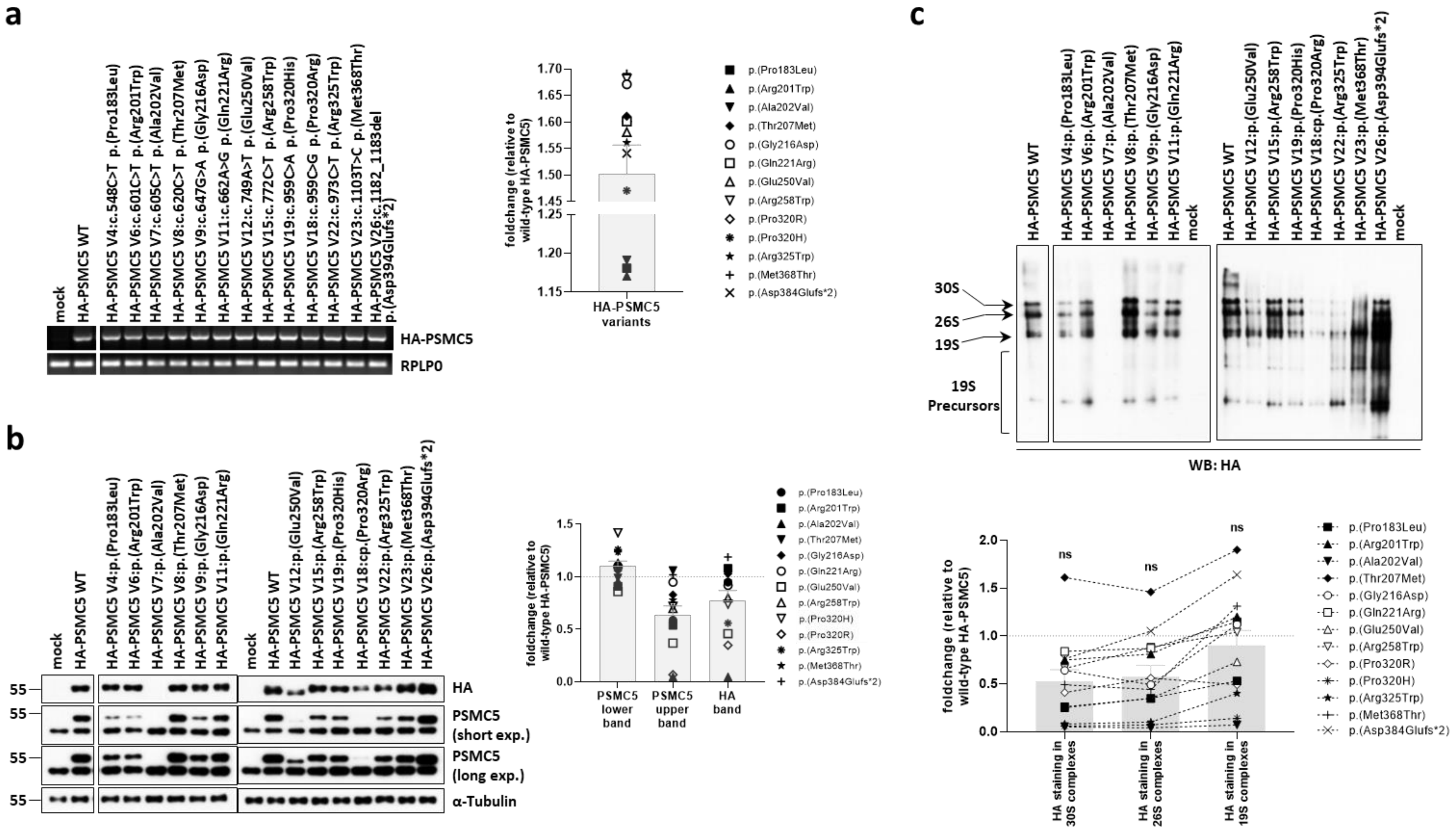

Supplementary Fig. 6: *PSMC5* variants differentially affect protein turnover and proteasome assembly in SHSY5Y cells. a SHSY5Y cells were transfected for 24 h with wild-type or mutant HA-tagged PSMC5 constructs. Semi-quantitative RT-PCR was performed using a BGH reverse primer to exclude endogenous transcripts. Right panel: HA-PSMC5 PCR bands were quantified by densitometry analysis and normalized to those

of RPLP0. Data are presented as normalized foldchange mean values ± SD of the HA-*PSMC5* variants (n=13) relative to wild-type controls (values set to 1; grid line). **b** SDS-PAGE/western blot analysis of 10 µg RIPA-extracted whole-cell lysates expressing N-terminally HA-tagged PSMC5 mutants detected HA and PSMC5/Rpt6 using specific antibodies, and normalizing by GAPDH. Dual exposure time revealed variant-specific differences in steady-state expression of HA-tagged PSMC5/Rpt6, as shown by the relative intensities of upper immunoreactive bands corresponding to full-length protein products. Right panel: densitometric quantification of HA and PSMC5 bands (upper and lower) is shown as fold-change (mean ± SD, n = 13) relative to wild-type (values set to 1; grid line). **c** Native-PAGE and western blot of TSDG-extracted lysates (20 µg) assessed incorporation of HA-tagged PSMC5 variants into proteasome complexes. Membranes were probed with anti-HA antibody. Arrows indicated migration of PSMC5-containing 30S, 26S and free 19S complexes, while a bracket marked a set of faster-migrating 19S assembly intermediates. The efficient incorporation of wild-type HA-tagged PSMC5/Rpt5 into unbound 19S regulatory particle positions, single-capped (19S-20S or 26S) and double-capped (19S-20S-19S or 30S) proteasomes, was evidenced by three migrating HA species. The efficient incorporation of wild-type HA-tagged PSMC5/Rpt5 into 19S, 26S and 30S complexes was evidenced by three HA species migrating at the positions of unbound 19S regulatory particle as well as single- (i.e. 19S-20S or 26S) and double-capped (i.e. 19S-20S-19S or 30S) proteasomes. Lower panel: the HA immunoreactive bands in the 30S, 26S and 19S complexes were quantified by densitometry and presented as fold changes for each HA-PSMC5 variant versus wild-type HA-PSMC5 controls whose densitometric values were set to 1 (grid line). Columns represent the foldchange mean values ± SD of the 13 investigated HA-PSMC5 variants, as indicated.

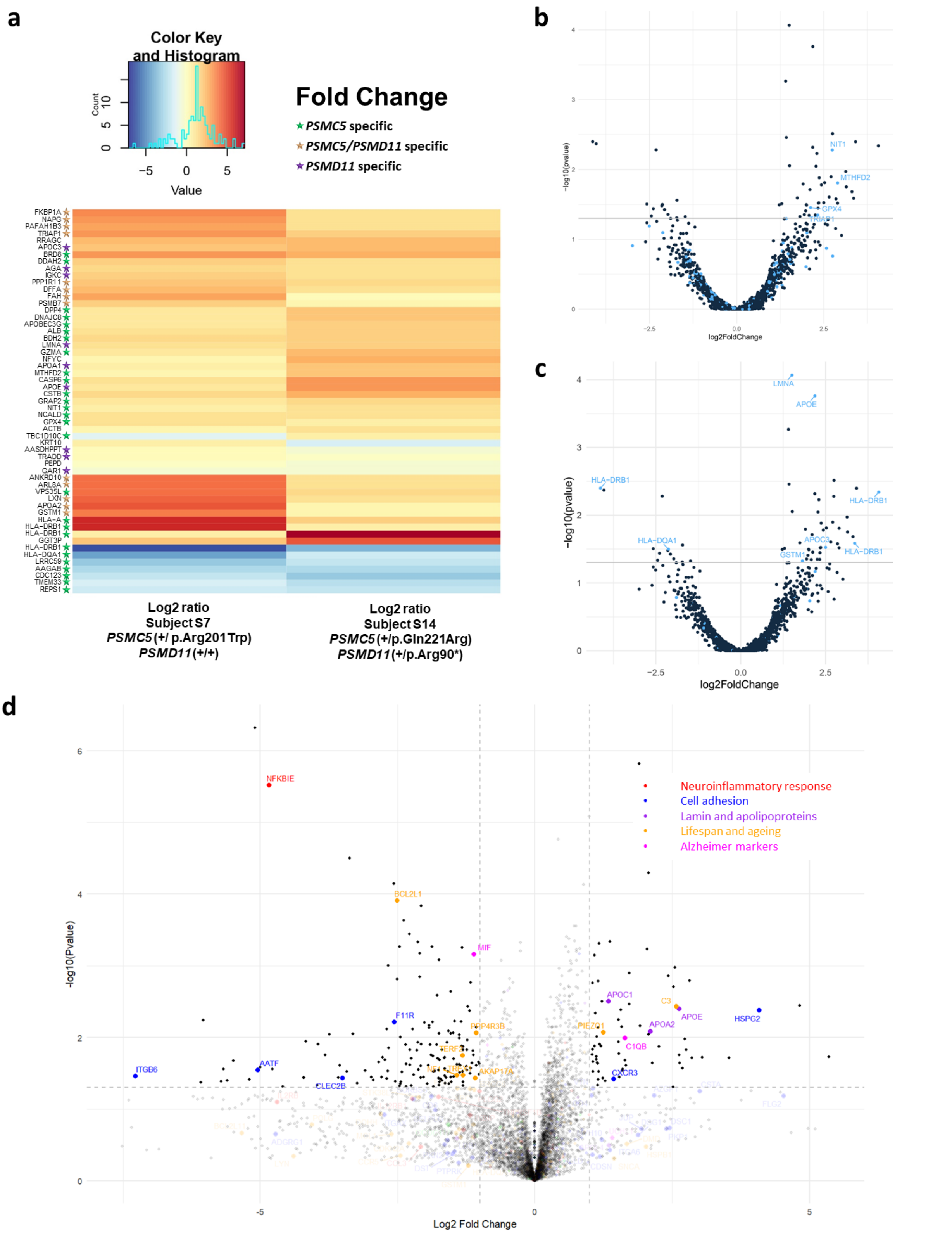

Supplementary Fig. 7: Proteome profiling of T cells from NDD subjects identifies candidate biomarkers for proteasome dysfunction. To decode the cellular effects of *PSMC5* loss-of-function causing NDD, total T-cell proteomes from affected subjects were subjected to two independent series of analyses by mass spectrometry proteomics and compared, using unaffected parents as controls. **a-c** In the first series, the proteomes of subjects S7 (p.Arg201Trp) and S14 (p.Gln221Arg) were compared with those of their respective mother and father. (**a**) On the left panel, heatmap of protein abundance pattern shows significantly altered proteins in S7 and S14 compared to parental controls, as indicated (p-value <0.05). Brackets indicate sets of proteins specifically altered in cells carrying *PSMC5*,

PSMD11 or a combination of *PSMC5* and *PSMD11* variants, as indicated. On the right upper panels, volcano plots show an enrichment in the deregulated expression of **(b)** genes encoding proteins localized in mitochondria and **(c)** genes encoding proteins reported deregulated with aging in literature. The blue points indicate the genes that are significantly deregulated ($p<0.05$). **d.** (Bottom panel) In the second series, the proteomes of subjects S14 (p.Gln221Arg), S15 (p.Glu250Val), S23 (p.Pro320Arg), S36 (p.Arg325Trp) and S37 (p.Arg325Trp) were compared with those of seven unrelated unaffected controls. Of note, samples from subjects S36 and S37, who harbor the same variant, were pooled in a single sample. The volcano plot shows the expression of genes encoding proteins significantly dysregulated ($p \leq 0.05$ and Fold Change > 2 or < -2). The colored dots correspond to genes encoding for proteins that belong to specific pathways significantly over- or under-enriched in subjects with NDD compared to controls.

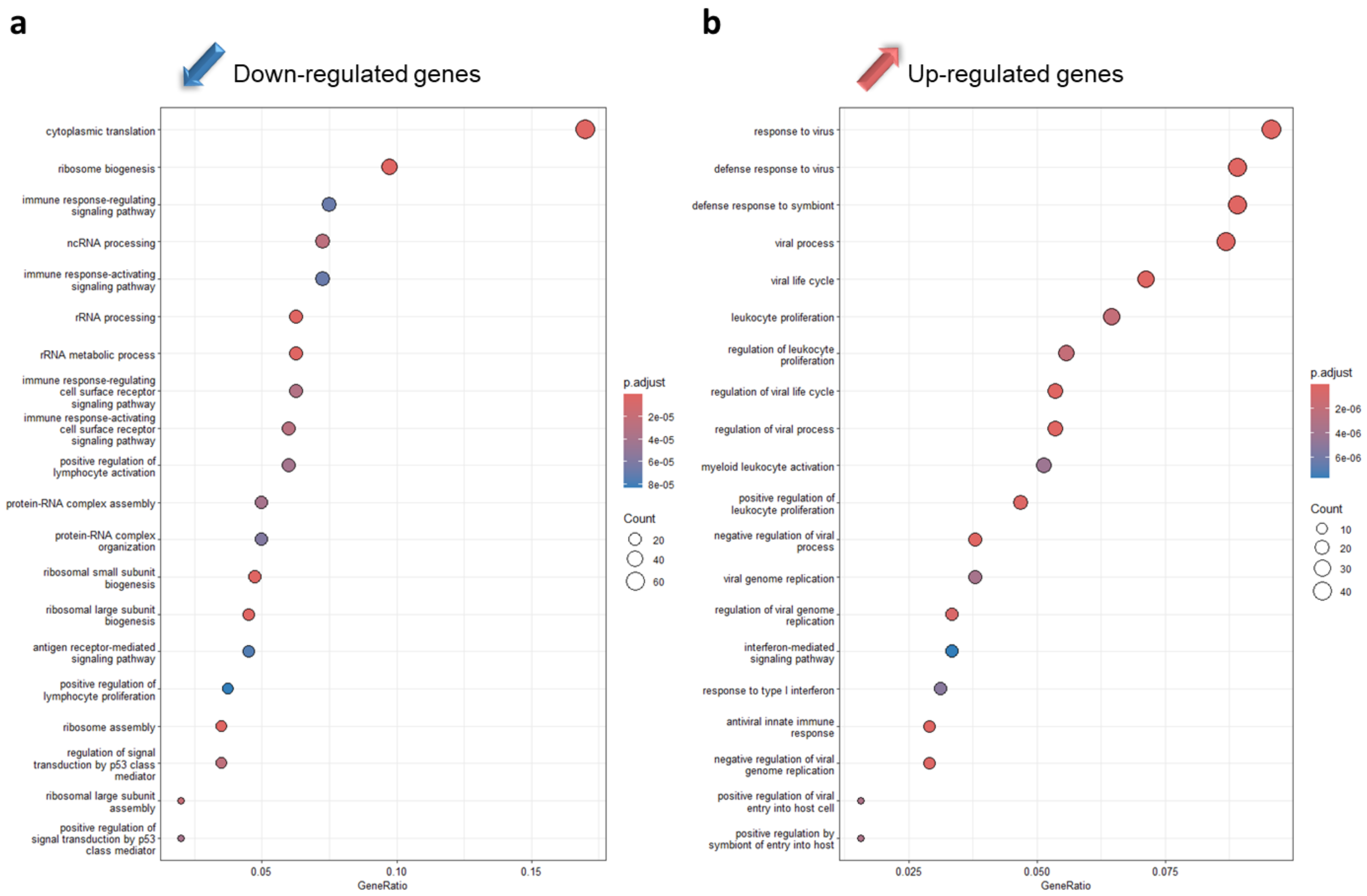

Supplementary Fig. 8: Transcriptomics of T cells from NDD subjects with *PSMC5* heterozygous variants confirms type I IFN response and suggests possible impairment of ribosome biogenesis. Transcriptomic analyses were performed in four affected individuals, namely subjects

S15 (p.Glu250Val), S23 (p.Pro320Arg), S36 (p.Arg325Trp) and S37 (p.Arg325Trp), and twelve unaffected unrelated controls. Dotplots show the main Gene Ontology (GO) terms for biological processes. **a** The significantly down-regulated genes are mostly associated with gene ontology (GO) terms related to impaired protein translation and, more specifically, to alterations in ribosome biogenesis. **b** Among the up-regulated genes, we observed an enrichment of those involved in antiviral response and, more specifically, in the response to type I IFN. The y-axis lists the enriched biological processes, while the x-axis represents the GeneRatio (proportion of genes associated with each term to the total number of down-regulated genes). The size of the dots corresponds to the number of genes involved in each process, and the color reflects the level of statistical significance (p.adjust), with redder shades indicating greater significance.

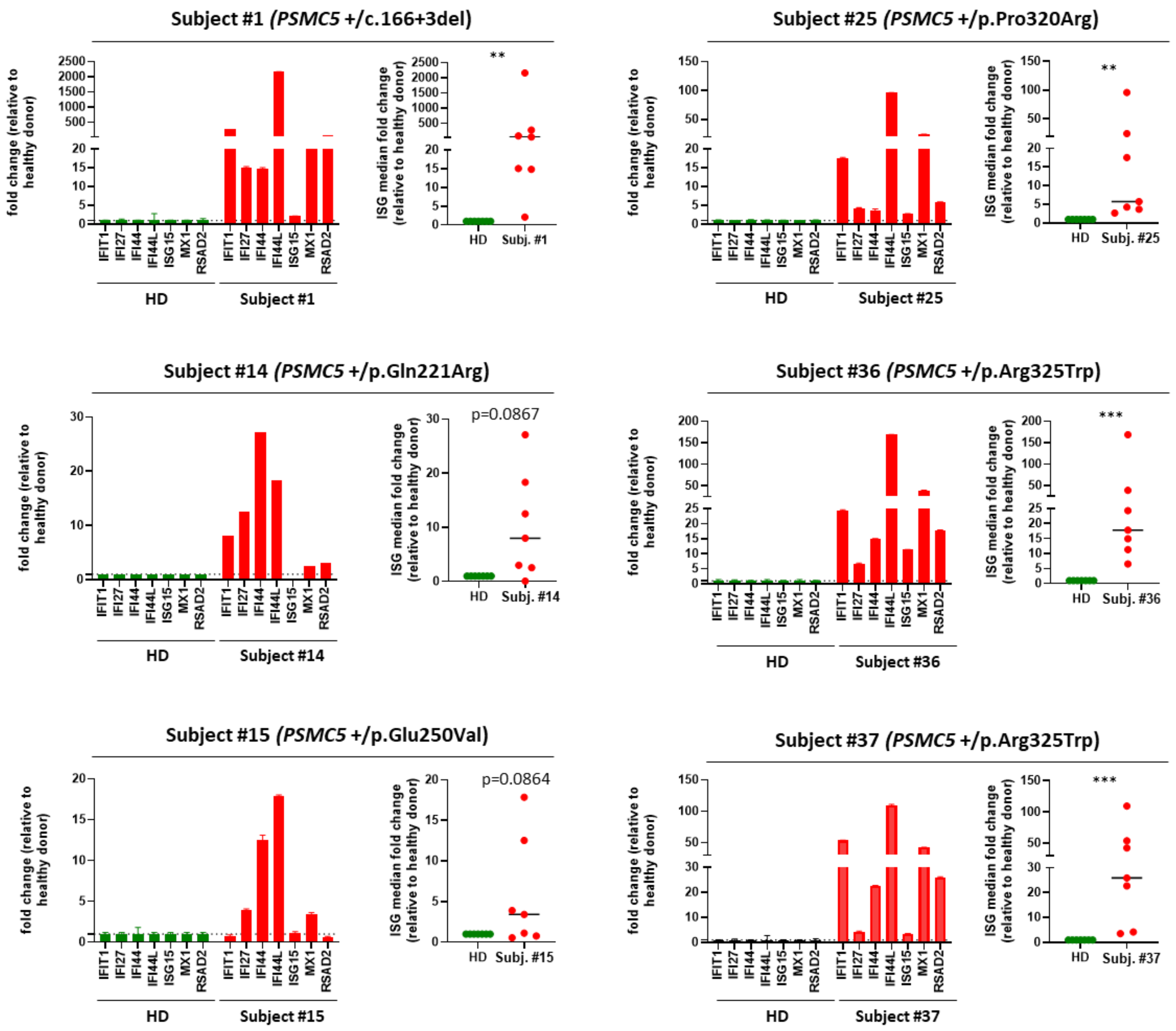

Supplementary Fig. 9: T cells from NDD subjects with *PSMC5* heterozygous variants exhibit a strong upregulation of ISG. Gene expression of seven typical ISG (*IFIT1*, *IFI27*, *IFI44*, *IFI44L*, *ISG15*, *MX1* and *RSAD2*) was assayed by RT-qPCR on T cells derived from NDD (n = 7) subjects S1 (c.166+3del p.?), S14 (p.Gln221Arg), S15 (p.Glu250Val), S23 (p.Pro320Arg), S36 (p.Arg325Trp) and S37 (p.Arg325Trp), as well as from healthy unrelated donors (HD; n = 7), as indicated. Expression levels were normalized to GAPDH and relative quantifications (RQ) are presented as fold change for each ISG (left) or fold change median for all ISG (right) over control T cells. Each ISG was considered as a biological replicate for the ISG median foldchange for each patient (**$p < 0.01$, ***$p < 0.001$, two-tailed ratio paired t-test). In more details the exact statistical values were: Subject S1: P=0.0035, t(6)=4.656; S14: P=0.0867, t(6)=2.046; S15: P=0.0864, t(6)=2.049; S25: P=0.0035, t(6)=4.729; S36: P=0.0002, t(6)=7.899; S37: P=0.0007, t(6)=6.323.

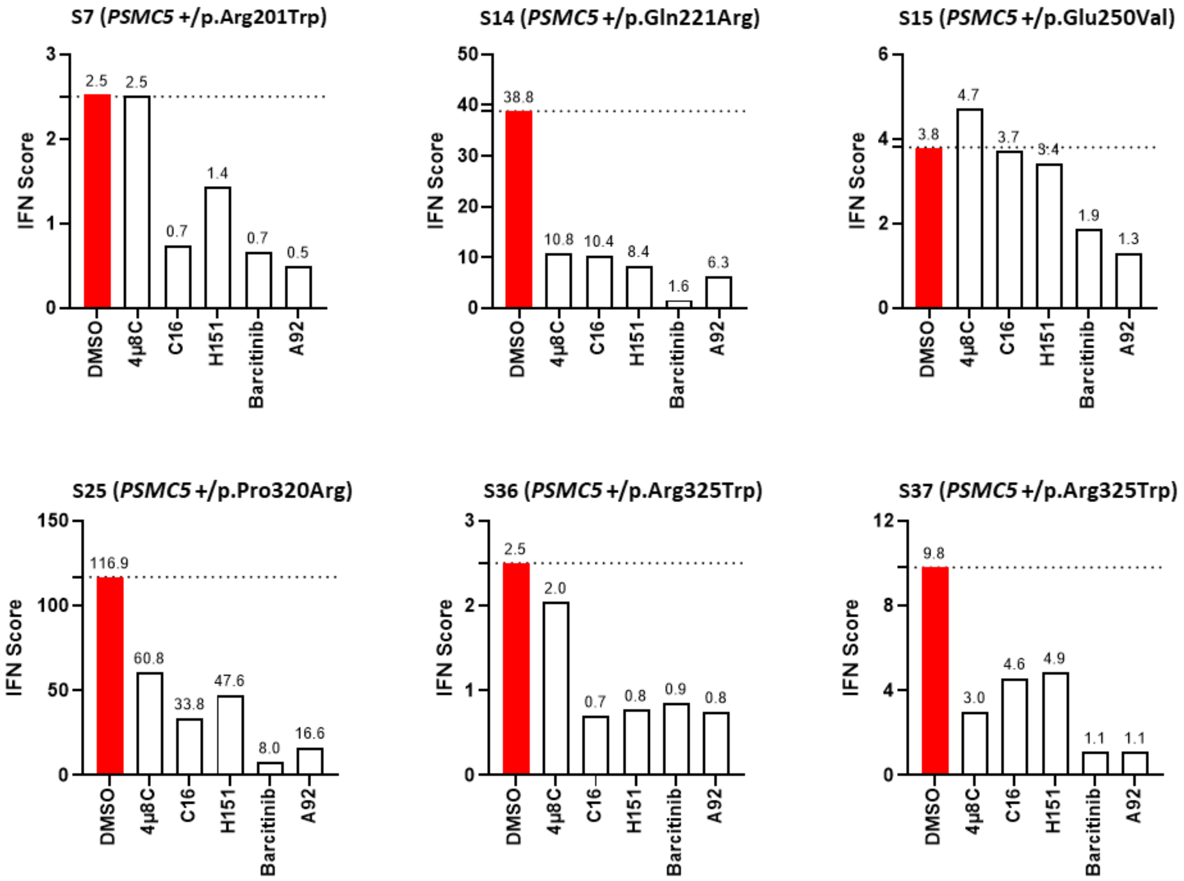

Supplementary Fig. 10: Sterile type I interferon (IFN) responses triggered in NDD patients with *PSMC5* heterozygous variants predominantly rely on the ISR. T cells expanded from PBMC isolated from NDD subjects S7, S14, S15, S25, S36 and S37 were subjected to an 8-h treatment with DMSO (vehicle, marked in red), 4µ8C (100 µM), C16 (3 µM), H-151 (2 µM), baricitinib (1 µM) or A92 (10 µM) prior to RNA extraction and RT-qPCR for expression analysis of *IFIT1*, *IFI27*, *IFI44*, *IFI44L*, *ISG15*, *MX1* and *RSAD2*. Shown are the IFN scores calculated for each T cell sample under different treatment conditions.

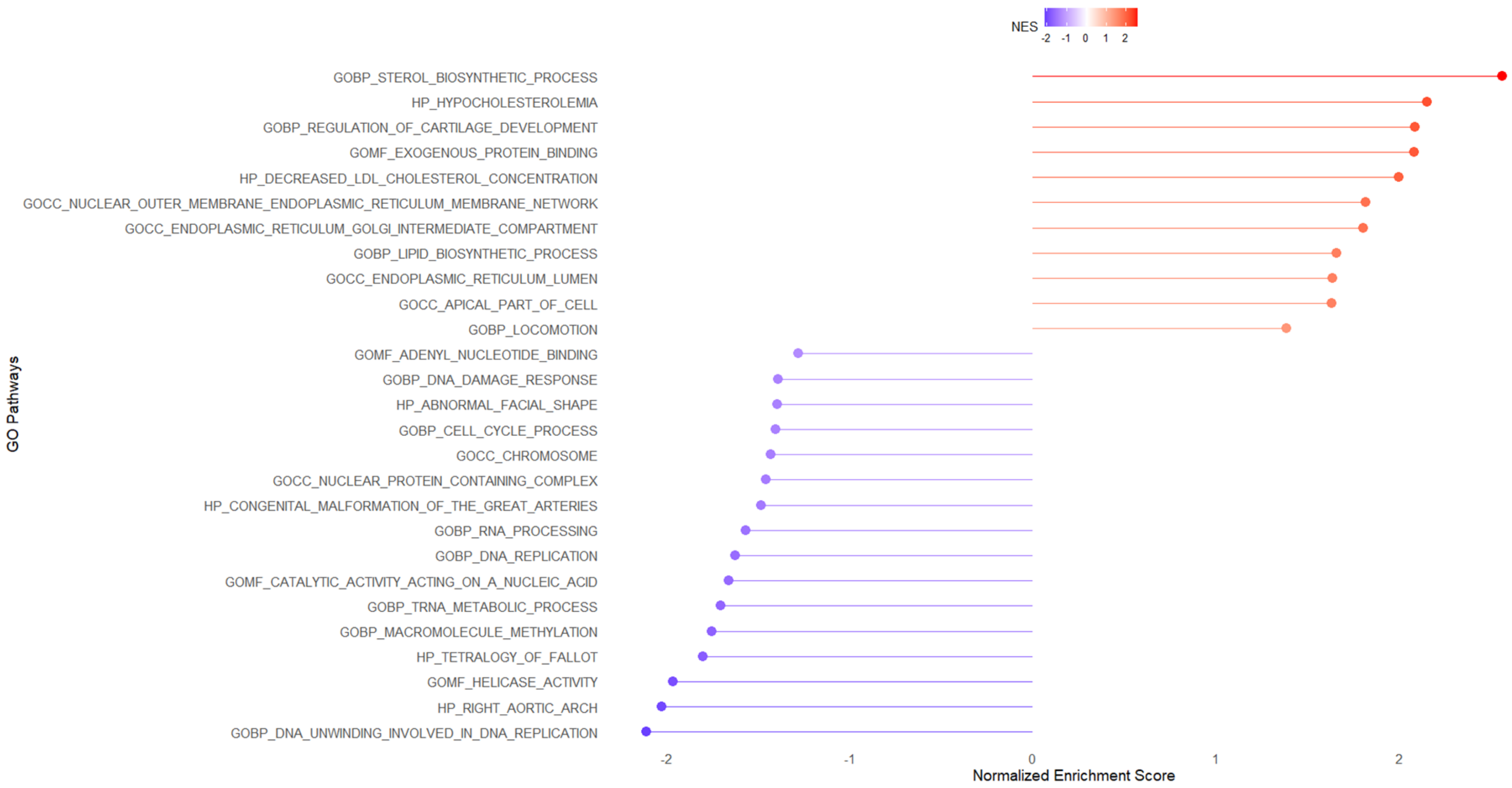

Supplementary Fig. 11: Transcriptomics of ectodermal cells derived from PSMC5$^{(+/p.Arg325Trp)}$ iPSCs highlights altered sterol and lipid metabolism and impairment of the process related to the development of craniofacial and conotroncal structures. The lollipop chart illustrates the deregulated processes determined by gene set enrichment analysis of the transcriptomic data. In purple are represented the down-regulated processes and pathways, whereas the up-regulated ones are represented in orange. The processes are ranked according to normalize enrichment scores.

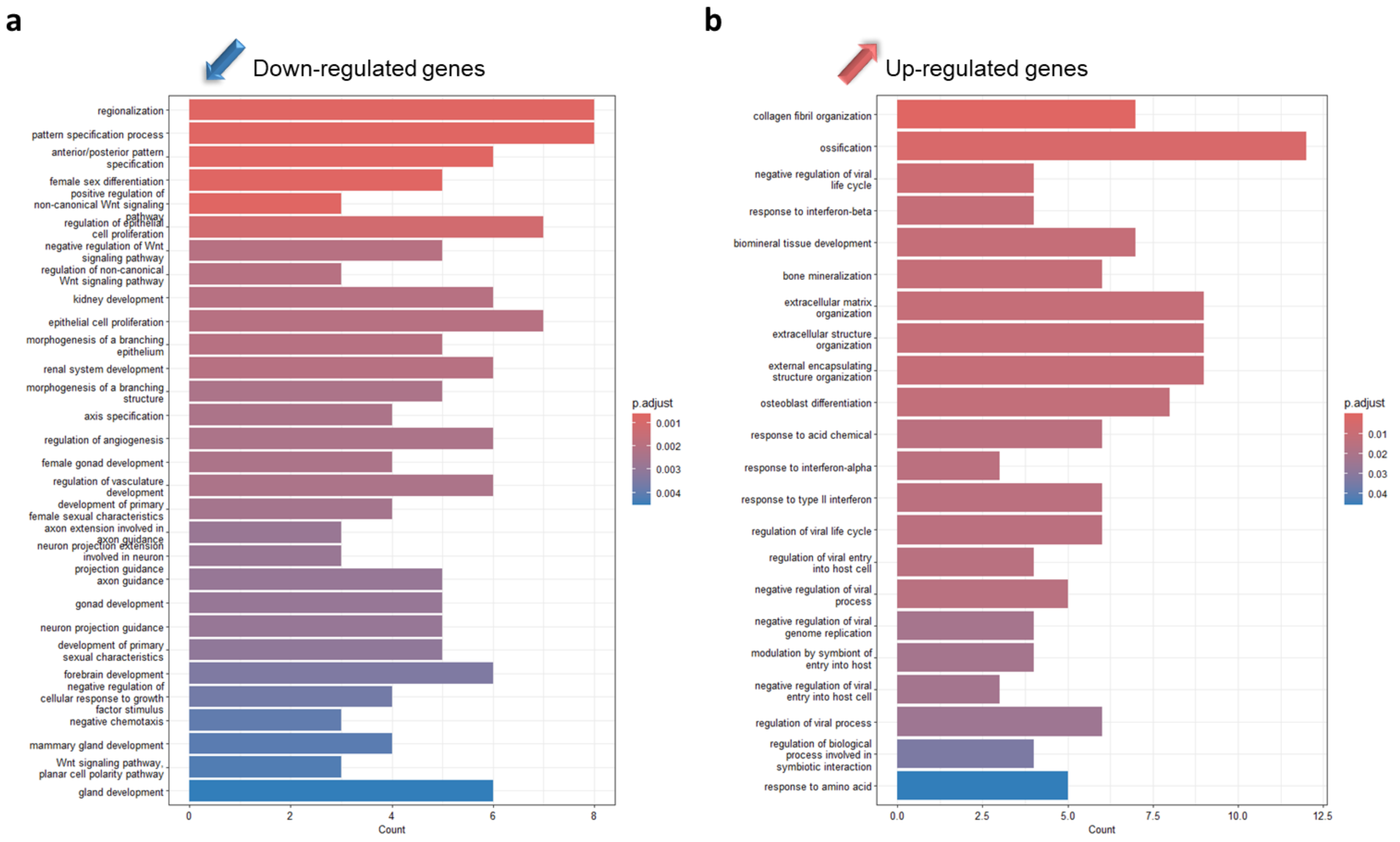

Supplementary Fig. 12: Transcriptomics of mesodermal cells derived from PSMC5$^{(+/p.Arg325Trp)}$ iPSCs highlights increased viral/interferon response and altered developmental processes. The bar plot shows the main Gene Ontology (GO) terms for biological processes enriched among **a** down-regulated genes, and **b** up-regulated genes. The size of the bars corresponds to the number of genes involved in each process (x-axis, "Count"), and the color reflects the level of statistical significance (p.adjust), with redder shades indicating greater significance. Biological processes are ranked according to adjusted *p* value.

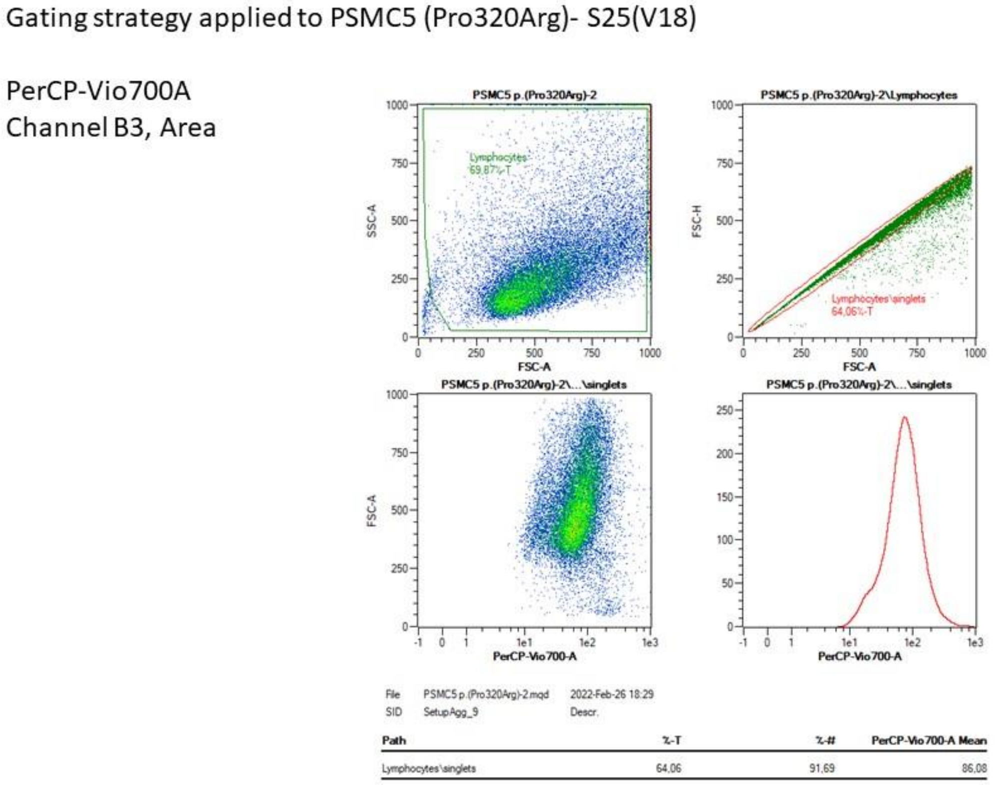

Supplementary Fig. 13: Example of the gating strategy used for analyses by flow cytometry. The quantification of aggresomes in patient T cells using the PROTEOSTAT® dye required the implementation of a gating strategy for flow cytometry analysis. More specifically, we used the B3 channel of the MACSQuant Analyzer that is suited for the detection of the fluorescence emitted by fluorochrome PerCP-Vio 700 (655-730 nm). In the figure, the T cells analyzed came from the affected individual S25 (variant p.(Pro320Arg)).

Supplementary references

1. Charpentier E*, et al.* 3’ RNA sequencing for robust and low-cost gene expression profiling.) (2021).

2. Letellier T*, et al.* Time-Limited Therapy with Belatacept in Kidney Transplant Recipients. *J Clin Med* **11**, (2022).

3. Chaumette T*, et al.* Monocyte Signature Associated with Herpes Simplex Virus Reactivation and Neurological Recovery after Brain Injury. *Am J Respir Crit Care Med* **206**, 295-310 (2022).

4. Menoret A, Agliano F, Karginov TA, Karlinsey KS, Zhou B, Vella AT. Antigen-specific downregulation of miR-150 in CD4 T cells promotes cell survival. *Frontiers in immunology* **14**, 1102403 (2023).

5. Martin M. Cutadapt removes adapter sequences from high-throughput sequencing reads. *EMBnetjournal* **17**, 10 (2011).

6. Li H, Durbin R. Fast and accurate short read alignment with Burrows-Wheeler transform. *Bioinformatics* **25**, 1754-1760 (2009).

7. Palma Medina LM*, et al.* Metabolic Cross-talk Between Human Bronchial Epithelial Cells and Internalized Staphylococcus aureus as a Driver for Infection. *Molecular & cellular proteomics : MCP* **18**, 892-908 (2019).

8. Suomi T, Elo LL. Enhanced differential expression statistics for data-independent acquisition proteomics. *Scientific reports* **7**, 5869 (2017).

9. Phipson B, Lee S, Majewski IJ, Alexander WS, Smyth GK. Robust Hyperparameter Estimation Protects against Hypervariable Genes and Improves Power to Detect Differential Expression. *The annals of applied statistics* **10**, 946-963 (2016).

10. Perez-Riverol Y*, et al.* The PRIDE database resources in 2022: a hub for mass spectrometry-based proteomics evidences. *Nucleic Acids Res* **50**, D543-D552 (2022).

11. Rath S*, et al.* MitoCarta3.0: an updated mitochondrial proteome now with sub-organelle localization and pathway annotations. *Nucleic Acids Res* **49**, D1541-D1547 (2021).

12. Pedrero-Prieto CM, Frontinan-Rubio J, Alcain FJ, Duran-Prado M, Peinado JR, Rabanal-Ruiz Y. Biological Significance of the Protein Changes Occurring in the Cerebrospinal Fluid of Alzheimer's Disease Patients: Getting Clues from Proteomic Studies. *Diagnostics (Basel)* **11**, (2021).

13. Van Dongen S. Graph Clustering Via a Discrete Uncoupling Process. *SIAM Journal on Matrix Analysis and Applications* **30**, 121-141 (2008).

14. Liu CW*, et al.* ATP binding and ATP hydrolysis play distinct roles in the function of 26S proteasome. *Molecular cell* **24**, 39-50 (2006).

15. Pautasso S*, et al.* Strategy of Human Cytomegalovirus To Escape Interferon Beta-Induced APOBEC3G Editing Activity. *J Virol* **92**, (2018).

16. Steffen J, Seeger M, Koch A, Kruger E. Proteasomal degradation is transcriptionally controlled by TCF11 via an ERAD-dependent feedback loop. *Molecular cell* **40**, 147-158 (2010).

17. Küry S, *et al.* De Novo Disruption of the Proteasome Regulatory Subunit PSMD12 Causes a Syndromic Neurodevelopmental Disorder. *Am J Hum Genet* **100**, 689 (2017).

18. Isidor B, *et al.* Stankiewicz-Isidor syndrome: expanding the clinical and molecular phenotype. *Genet Med* **24**, 179-191 (2022).

19. Ebstein F, *et al.* PSMC3 proteasome subunit variants are associated with neurodevelopmental delay and type I interferon production. *Sci Transl Med* **15**, eabo3189 (2023).

20. Deb W, *et al.* PSMD11 loss-of-function variants correlate with a neurobehavioral phenotype, obesity, and increased interferon response. *Am J Hum Genet* **111**, 1352-1369 (2024).

21. Cuinat S, *et al.* Understanding neurodevelopmental proteasomopathies as new rare disease entities: A review of current concepts, molecular biomarkers, and perspectives. *Genes Dis* **11**, 101130 (2024).

22. Ansar M, *et al.* Biallelic variants in PSMB1 encoding the proteasome subunit beta6 cause impairment of proteasome function, microcephaly, intellectual disability, developmental delay and short stature. *Human molecular genetics*, (2020).

23. Kroll-Hermi A, *et al.* Proteasome subunit PSMC3 variants cause neurosensory syndrome combining deafness and cataract due to proteotoxic stress. *EMBO Mol Med* **12**, e11861 (2020).

24. Papendorf JJ, *et al.* Identification of eight novel proteasome variants in five unrelated cases of proteasome-associated autoinflammatory syndromes (PRAAS). *Frontiers in immunology* **14**, 1190104 (2023).

25. Zhang S, *et al.* USP14-regulated allostery of the human proteasome by time-resolved cryo-EM. *Nature* **605**, 567-574 (2022).

26. Dong Y, *et al.* Cryo-EM structures and dynamics of substrate-engaged human 26S proteasome. *Nature* **565**, 49-55 (2019).
