# Supplementary material for: Investigating the neuronal role of the proteasomal ATPase subunit gene PSMC5 in neurodevelopmental proteasomopathies
Source: Nat Commun. 2025 Nov 26;16:10545. doi: 10.1038/s41467-025-65556-8 (PMC12658096; doi:10.1038/s41467-025-65556-8)
Supplement: Supplementary file 2 — Description of Additional Supplementary Files [file 41467_2025_65556_MOESM2_ESM.pdf]

Description of Additional Supplementary Files

Supplementary Table 1: Predictions and functional tests for the PSMC5 variants identified in the affected individuals included in the study.

Supplementary Table 2: Clinical features of the subjects with *PSMC5* variants and indels.

Supplementary Table 2a: Detailed clinical features of the subjects with *PSMC5* variants and indels.

Supplementary Table 2b: Clinical features described by Human Phenotype Ontology (HPO) terms across the patient cohort.

Supplementary Table 2c: Individual lists of symptoms and signs observed in each affected subjects of the study.

Supplementary Table 3: First series of proteomics analyses.

Supplementary Table 3a: LC-MS/MS parameter (data dependent mode, spectral library).

Supplementary Table 3b: LC-MS/MS parameter (data independent mode; quantitative data).

Supplementary Table 3c: Peptide and protein identification parameters in Spectronaut.

Supplementary Table 3d: Results of proteome-wide analysis indicated by Hi3 peptide intensity data: comparison at the family level between individual data from affected individuals S6 and S11 and individual data from their parental controls.

Supplementary Table 3e: Results of proteome-wide analysis indicated by Hi3 peptide intensity data: comparison between combined data from affected individuals S6 and S11 and combined data from their parental controls.

Supplementary Table 4: Datasets of genes used for functional enrichment analysis from proteomics and transcriptomics data.

Supplementary Table 4a: List of genes related to aging used for functional enrichment analysis from proteomics and transcriptomics data.

Supplementary Table 4b: List of genes suggested as biomarkers of Alzheimer's disease in cerebrospinal fluid by Pedredo-Prieto *et al.* used for functional enrichment analysis from proteomics and transcriptomics data.

Supplementary Table 5: Materials used in this study.

Supplementary Table 5a: Reagents, software, and equipment used in this study.

Supplementary Table 5b: Oligonucleotide sequences used in this study.

Supplementary Table 5c. Animal strains used in this study.

Supplementary Table 5d. Cell lines used in this study.

Supplementary Table 5e. Web resources for prediction of variant pathogenicity and 3D modeling.

Supplementary Table 6. List of participating teams and ethics committees.

Supplementary Table 6a. List of diagnostic or research teams involved in this study.

Supplementary Table 6b. List of local ethics committees that approved subject inclusion and sample collection.
