# Supplementary material for: Investigating the neuronal role of the proteasomal ATPase subunit gene PSMC5 in neurodevelopmental proteasomopathies
Source: Nat Commun. 2025 Nov 26;16:10545. doi: 10.1038/s41467-025-65556-8 (PMC12658096; doi:10.1038/s41467-025-65556-8)
Supplement: Supplementary file 9 — Reporting Summary [file 41467_2025_65556_MOESM9_ESM.pdf]

nature portfolio

Corresponding author(s): Sébastien KÜRY; Elke Krüger

Last updated by author(s): Sep 12, 2025

Reporting Summary

Nature Portfolio wishes to improve the reproducibility of the work that we publish. This form provides structure for consistency and transparency in reporting. For further information on Nature Portfolio policies, see our Editorial Policies and the Editorial Policy Checklist.

Statistics

For all statistical analyses, confirm that the following items are present in the figure legend, table legend, main text, or Methods section.

n/a	Confirmed	
☐	☒	The exact sample size (*n*) for each experimental group/condition, given as a discrete number and unit of measurement
☐	☒	A statement on whether measurements were taken from distinct samples or whether the same sample was measured repeatedly
☐	☒	The statistical test(s) used AND whether they are one- or two-sided<br>*Only common tests should be described solely by name; describe more complex techniques in the Methods section.*
☐	☒	A description of all covariates tested
☐	☒	A description of any assumptions or corrections, such as tests of normality and adjustment for multiple comparisons
☐	☒	A full description of the statistical parameters including central tendency (e.g. means) or other basic estimates (e.g. regression coefficient) AND variation (e.g. standard deviation) or associated estimates of uncertainty (e.g. confidence intervals)
☒	☐	For null hypothesis testing, the test statistic (e.g. *F*, *t*, *r*) with confidence intervals, effect sizes, degrees of freedom and *P* value noted<br>*Give P values as exact values whenever suitable.*
☒	☐	For Bayesian analysis, information on the choice of priors and Markov chain Monte Carlo settings
☒	☐	For hierarchical and complex designs, identification of the appropriate level for tests and full reporting of outcomes
☒	☐	Estimates of effect sizes (e.g. Cohen's *d*, Pearson's *r*), indicating how they were calculated

Our web collection on statistics for biologists contains articles on many of the points above.

Software and code

Policy information about availability of computer code

Data collection	No software was used
Data analysis	Only commercial or published software were used. It is provided in Supplementary Fig. 5a.<br>For 3D structural analysis of PSMC5 variants, we used UCSF ChimeraX (https://www.rbvi.ucsf.edu/chimerax/), which is free of charge for academic use.<br>For analysis of neuronal images in rat and moue, we used the open source software ImageJ v1.53 (https://imagej.net/ij/index.html). For analysis of mouse hippocampal neuron images, we added NeuronJ, which is an open source plugin of ImageJ.<br>For lipid profile analyse, we used LipidSearch™ 4.1.16 Software (version no longer commercialized).<br>For transcriptomic analyses, we used open source softwares R packages DESeq2 (https://bioconductor.org/packages/devel/bioc/html/DESeq2.html), clusterProfiler (https://www.bioconductor.org/packages/release/bioc/html/clusterProfiler.html) and ggplot2 (https://cran.r-project.org/web/packages/ggplot2/index.html).<br>For variant analyses, we also used Combined Annotation Dependent Depletion, CADD (https://cadd.gs.washington.edu/), dbSNP (http://www.ncbi.nlm.nih.gov/projects/SNP/, DRSC Integrative Ortholog Prediction Tool, DIOPT (https://www.flyrnai.org/cgi-bin/DRSC_orthologs.pl), GeneMatcher (https://genematcher.org/), gnomAD (http://gnomad.broadinstitute.org/), InterVar (https://wintervar.wglab.org/), Metadome (https://stuart.radboudumc.nl/metadome/), Missense Tolerance Ratio Gene Viewer (http://mtr-viewer.mdhs.unimelb.edu.au/), and 3D Missense Tolerance Ratio, MTR3D (https://biosig.lab.uq.edu.au/mtr3d/), MobiDetails (https://mobidetails.iurc.montp.inserm.fr/MD/).

For manuscripts utilizing custom algorithms or software that are central to the research but not yet described in published literature, software must be made available to editors and reviewers. We strongly encourage code deposition in a community repository (e.g. GitHub). See the Nature Portfolio guidelines for submitting code & software for further information.

Data

Policy information about availability of data

All manuscripts must include a data availability statement. This statement should provide the following information, where applicable:

- Accession codes, unique identifiers, or web links for publicly available datasets
- A description of any restrictions on data availability
- For clinical datasets or third party data, please ensure that the statement adheres to our policy

All data supporting the findings of this study are available within the Article and its Supplementary Information files. Source data underlying the figures are provided in the accompanying Source Data file. The Source Data file includes raw measurements, quantified values, and uncropped scans of gels and blots used to generate the main figures and Supplementary Figs. Mass spectrometry proteomics data have been deposited to the via in the PRIDE repository through the ProteomeXchange Consortium under accession codes PXD048558 and PXD058728 (https://www.ebi.ac.uk/pride/archive/projects/PXD048558; https://www.ebi.ac.uk/pride/archive/projects/PXD058728). Lipidomics data have deposited in a repository of the Metabolomics Workbench with the dataset identifier http://dx.doi.org/10.21228/M8TK06. Transcriptomics and Nanostring data have been deposited in the Gene Expression Omnibus (GEO) under respective accession codes GSE288665 and GSE306813 (https://www.ncbi.nlm.nih.gov/geo/query/acc.cgi?acc=GSE288665; https://www.ncbi.nlm.nih.gov/geo/query/acc.cgi?acc=GSE306813).

Research involving human participants, their data, or biological material

Policy information about studies with human participants or human data. See also policy information about sex, gender (identity/presentation), and sexual orientation and race, ethnicity and racism.

Reporting on sex and gender	We obtained the consent of the participants or their legal guardians to indicate the sex of the affected individuals reported. However, since individual data potentially identifying had to be removed, we provided the information in the form of percentages of female and male patients (Fig. 2a). .This data is of scientific interest as the increased number of males (60% males, 40% females) is in line with the observations made in general for neurodevelopmental disorders.<br>On the other hand, we did not report on gender.
Reporting on race, ethnicity, or other socially relevant groupings	The PSMC5-associated neurodevelopmental disorder can virtually affect all individuals, disregarding of their ethnic or social group. We therefore did not ask clinicians and families for this information, as these are not confounding factors.
Population characteristics	The study population is pediatric, since PSMC5 variants impair pre- and post-natal development. We therefore report on the age and genotypic information related to the participants, as well as on their clinical symptoms and signs that could allow us to understand the pathophysiological impact of the variants. Previous diagnostic hypotheses, other possible genetic finding or treatment were asked to the clinicians and the families, whenever relevant.
Recruitment	The participants were recruited on the results of the genetic investigations performed on a diagnostic or research setting to identify the molecular cause of the disorder. The affected individuals were identified either through direct connections with the participating clinical teams, or through the exchange platform GeneMatcher or Decipher. Since the findings reported in these platforms mostly relate to a pediatric population with severe phenotypes, we may have missed distinct and milder phenotypes in adults. This could be further explored in follow up studies.
Ethics oversight	All data included in this study were collected and shared in compliance with institutional policies and applicable regulations. Centers enrolling patients in a research context obtained approval from their local ethics committees (Supplementary Table 6b). For selected centers contributing data from a single patient in a diagnostic setting, IRB approval was not required, as the data were fully anonymized prior to transfer and excluded any photographic or personally identifiable information.

Note that full information on the approval of the study protocol must also be provided in the manuscript.

Field-specific reporting

Please select the one below that is the best fit for your research. If you are not sure, read the appropriate sections before making your selection.

☒ Life sciences ☐ Behavioural & social sciences ☐ Ecological, evolutionary & environmental sciences

For a reference copy of the document with all sections, see nature.com/documents/nr-reporting-summary-flat.pdf

Life sciences study design

All studies must disclose on these points even when the disclosure is negative.

Sample size	Due to the rarity of the disorder, the sample size could not be determined beforehand. It depended on the willingness of families to contribute to the study by providing samples. Statistical methods were not relevant in this case, as the main factor determining sample size was human.<br>1. Experiments on T cells: it could not be calculated or anticipated, as the enrollment of affected individuals depended entirely on the identification of the variants by geneticists working virtually all over the world, and for testing T cells, it depended on the participants consenting to provide blood samples; 2. Assays on Drosophila: No sample size calculation was performed. Sample sizes were chosen based on a long established and published protocol; 3. Neuronal transfection experiments: For every condition a minimum of 8 neurons were imaged

	per batch, for a minimum of 2 independent primary hippocampal neuron batches. Minimal 8 neurons per batch due to the neuronal morphology variability, and minimal 2 neuronal batches for technical differences. Extensive experience from past experiments have shown this n gives sufficient power to find significant differences; 4. Proteomics: sample size for the two round of analyses was determined by the patient material available; 5. Transcriptomics: sample size for the two rounds of analyses was determined by the patient material available; 6. Lipidomics: Sample size was determined on samples provided by the collaborator. In detail, 13 lipid samples were analyzed (7 patient, 6 relative samples)
Data exclusions	1. Experiments on T cells: no data were excluded. All the families (unaffected parents) and their affecting members who consented to provide blood samples were included ; 2. Assays on Drosophila: No data was excluded; 3. Neuronal transfection experiments: No data was excluded as with neuronal morphology there are no clear criteria to exclude data. Only dead neurons were not taken along for morphological analysis; 4. Proteomics: No data were excluded; 5. Transcriptomics: No data were excluded; 6. Lipidomics: No data wer excluded.
Replication	For all the experiments proposed, we used biological replicates and no technical replicates, as specified in the figure legends. Specifically: 1. Experiments on T cells: all the experiments were duplicated or triplicated; 2. Assays on Drosophila: Repeated measures over multiple days were collected and combined. This entire process was then replicated at a later date and yielded the same result. 3. Neuronal transfection experiments: All conditions have at least been tested in 2 different batches, which can be seen as biological replicates; 4. Proteomics: Two rounds of independent analyses performed on distinct platforms were performed. 4.1. For the first round, two patients (S7 and S14) were analyzd against two unaffected controls, using two bioreplicates for each individual. In the second round, for lysate analysis, five patients with PSMC5 variants were analyzed against seven control patients. 4.2. For the anti-alpha2 immunopurifications, five patients were analyzed againt 4 control patients and each experiment was performed and analyzed in triplicate. For statistical analysis, t-test were used with p-values <0.05. Data from the second round analyses were used to validate the results obtained with the first round of analysis; 5. Transcriptomics: Transcriptomics analysis was performed on two cell types: (i) Lymphocytes T cells in two technical replicates : twelve control samples from unaffected, unrelated individuals and four samples from subjects with pathogens variants in PSMC5 S15 (p.Glu250Val), S23 (p.Pro320Arg), S36 (p.Arg325Trp) and S37 (p.Arg325Trp); (ii) Ectodermal cells differentiated : three derived from the iPSc clones harboring the variant p.(Arg325Trp) in PSMC5, two derived from the isogenic control WT iPSc clones and one derived from the commercial iPSc line ASE-9211; 6. Lipidomics: Each patient/relative sample was injected twice per polarity for the lipidomics LC-MSMS measurements.
Randomization	1. Experiments on T cells: not applicable; 2. Assays on Drosophila: The allocation of groups was based on the genetic cross desired. The same fruit fly line sources were used as parents for the different genetic crosses with allocation of parents from a genotype being random. 3. Neuronal transfection experiments: not relevant as all transfections were done in neurons coming from wildtype mice and we can only transfect 1 construct per neuronal well; 4. Proteomics: n/a; 5. Tanscriptomics: For sample processing, T lymphocytes were randomized to account for individual variability and minimize batch effects. In contrast, ectodermal cells were not randomized, as their differentiation process was strictly controlled and lineage-dependent, limiting external variability; 6. Lipidomics: Samples were measured in a randomized order.
Blinding	The investigators were not blinded for the study. Indeed, given the rarity of the disorder, and therefore the limited number of biolological samples were rare and the study, added to the exploratory nature of the study, the investigators had to know the distribution of the groups so as to be able to plan the collection and analysis of data effectively. A blinding protocol would have implied a staff reorganization, which was not in line with the size and resources of the team. It would have thus increased the risk of obtaining results of lower scientific quality. In details:<br>1. Experiments on T cells: n/a; 2. Assays on Drosophila: Blinding was not performed; 3. Neuronal transfection experiments: The experimenter that analysed the morphology was blind for the condition during analysis; 4. Proteomics: n/a; 5. Transcriptomics: n/a; 6. Lipidomics: Blinding was not performed.

Reporting for specific materials, systems and methods

We require information from authors about some types of materials, experimental systems and methods used in many studies. Here, indicate whether each material, system or method listed is relevant to your study. If you are not sure if a list item applies to your research, read the appropriate section before selecting a response.

Materials & experimental systems

n/a	Involved in the study	
☐	☒	Antibodies
☐	☒	Eukaryotic cell lines
☒	☐	Palaeontology and archaeology
☐	☒	Animals and other organisms
☐	☒	Clinical data
☒	☐	Dual use research of concern
☒	☐	Plants

Methods

n/a	Involved in the study	
☒	☐	ChIP-seq
☐	☒	Flow cytometry
☒	☐	MRI-based neuroimaging

Antibodies

Antibodies used	All the antibodies used in the study are listed in Supplementary Table S5a.<br>1. Experiments on T cells and SH-SY5Y cell lines: Antibodies employed in this study comprised HA (BioLegend, clone HA.11), PSMC5/Rpt6 (Enzo Life Sciences, clone p45-110), α6 (Enzo Life Sciences, clone MCP20), ubiquitin K48-linked ubiquitin chains (Cell Signaling, clone D9D5), GAPDH (Cell Signaling, clone 14C10), and beta-actin (Santa Cruz Biotechnology, clone C4); 2. Neuronal transfection experiments: MAP2 (1:500, Synaptic System; #188004); donkey-anti-guinea pig Alexa647 (1:200; Jackson ImmunoResearch #706-605-148); Glut (1:1000, Synaptic Systems #135304); vGAT (1:200, Synaptic System #131003); Ubiquitin polyclonal Ab (1:500, ThermoFisher Scientific #PA1-10023); PSMC5 (1:400, Sigma-Aldrich); 3. Assays on iPSC-derived lines: SOX2 (Synaptic System, Cat.No

	347 003); Nestin (Synaptic System, Cat.No 312 011); indirect immunofluorescence was performed using Alexa Fluor® 488-conjugated goat anti-rabbit IgG (1:1,000, Invitrogen, A11094) and Alexa Fluor® 568-conjugated donkey anti-mouse IgG (1:1,000, Invitrogen, A10037).
Validation	We have provided a link for the relevant data sheets for each antibody in Supplementary Table S5a. The data sheet includes the manufacturer's validation statements, quality control procedures and relevant citations. In more details:<br>1. Experiments on T cells and SH-SY5Y cell lines: The HA and PSMC5/Rpt6 antibodies were validated through overexpression experiments (Fig. S6). The specificity of the anti-ubiquitin K48-linked ubiquitin chains antibody was confirmed by its capacity to react specifically with purified K48-linked ubiquitin chains, as described by the manufacturer (Cell Signaling, product information: https://www.cellsignal.com/products/primary-antibodies/k48-linkage-specific-polyubiquitin-antibody/4289). Validation of the anti-alpha6 antibody was established by its ability to interact with purified proteasomes (PMID: 2882161). The anti-GAPDH antibody underwent validation by the manufacturer through immunohistochemical experiments in the presence of control and antigen-specific peptides, ensuring its specificity and reliability (Cell Signaling, product information: https://www.cellsignal.com/products/primary-antibodies/gapdh-14c10-rabbit-mab/2118). Lastly, the specificity of the anti-beta actin antibody was confirmed by the manufacturer through its capability to control siRNA-mediated downregulation of beta-actin (Santa Cruz Biotechnology, product information: https://datasheets.scbt.com/sc-47778.pdf); 2. Neuronal transfection experiments: For all validated studies on the manufacturer's website: MAP2: https://sysy.com/product/188004; Alexa647: https://www.jacksonimmuno.com/catalog/products/706-605-148; vGlut1: https://sysy.com/product/135304, vGAT: https://sysy.com/product/131003#list, Ubiquitin polyclonal Ab: https://www.thermofisher.com/antibody/product/Ubiquitin-Antibody-Polyclonal/PA1-10023, PSMC5: https://www.sigmaaldrich.com/IE/en/product/sigma/hpa017871. For our own validation, see for example: PLoS Biol. 2021 May 26;19(5):e3001279. doi: 10.1371/journal.pbio.3001279. eCollection 2021 May. PMID: 34038402 and Hum Mutat. 2021 Apr;42(4):445-459. doi: 10.1002/humu.24176. Epub 2021 Mar 1. PMID: 33565190; 3. For all validated studies on the manufacturer's website: SOX2: https://sysy.com/product/347003, Nestin: https://sysy.com/product/312011#list, Alexa Fluor® 488-conjugated goat anti-rabbit IgG: https://www.thermofisher.com/antibody/product/Alexa-Fluor-488-Antibody-Recombinant-Superclonal/710369, Alexa Fluor® 568-conjugated donkey anti-mouse IgG: https://www.thermofisher.com/antibody/product/Donkey-anti-Mouse-IgG-H-L-Highly-Cross-Adsorbed-Secondary-Antibody-Polyclonal/A10037

Eukaryotic cell lines

Policy information about cell lines and Sex and Gender in Research

Cell line source(s)	Details are provided in Supplementary Table S5d<br>1. The SH-SY5Y neuroblastoma cell line used in this study was obtained from the laboratory stock maintained at the Institute of Medical Biochemistry and Molecular Biology, University Medicine Greifswald, Germany; 2. Mouse primary hippocampal neurons came from male and female wildtype mouse embryos; 3. Rat hippocampal neurons were commercially available and purchased from Innoprot (RN-h, P10101); 3. All iPSC lines used for the study derived from the commercial iPSC line ASE-9211 purchased from Applied StemCell (Milpitas, USA via CliniSciences, France)
Authentication	1. SH-SY5Y: No authentification was performed; 2. Mouse neurons: We isolated and dissociated the neurons ourself; 3. rat neurons: the authentication was provided by Innoprot; 3. iPSC line ASE-9211: the authentication was provided by CliniSciences
Mycoplasma contamination	1. SH-SY5Y: The cell line underwent routine mycoplasma contamination checks; 2. Mouse neurons: No testing for mycoplasma was done; 3. Rat neurons: No testing for mycoplasma was done; 4. iPSC line: the cell line underwent routine mycoplasma contamination checks
Commonly misidentified lines<br>(See ICLAC register)	n/a

Animals and other research organisms

Policy information about studies involving animals; ARRIVE guidelines recommended for reporting animal research, and Sex and Gender in Research

Laboratory animals	1. Drosophila melanogaster: Drosophila melanogaster aged 1-3days old; 2. Mus musculus: FvB mice
Wild animals	The study did not involve wild animals.
Reporting on sex	1. Drosophila: Sex was not considered in the study design; 2. Mus musculus: male and female embryos were used and neurons were mixed as the hippocampus was isolated at E16.5
Field-collected samples	1. Drosophila: The study did not involve samples collected from the field; 2: Mus musculus: n/a
Ethics oversight	1. Drosophila: Ethics approval is not required for the use of invertebrates in research at the University of Alberta. Approved procedures were followed for biohazard management; 2. Mus musculus: All animal experiments were conducted in accordance with the European Commission Council Directive 2010/63/EU (CCD project license AVD101002017893), and all described experiments and protocols were subjected to ethical review (and approved) by an independent review board (IRB) of the Erasmus MC.

Note that full information on the approval of the study protocol must also be provided in the manuscript.

Clinical data

Policy information about clinical studies

All manuscripts should comply with the ICMJE guidelines for publication of clinical research and a completed CONSORT checklist must be included with all submissions.

Clinical trial registration	n/a
Study protocol	n/a
Data collection	Clinical data were collected, albeit not in the context of a clinical trial. After that the parents and/or legal guardians of the affected individuals gave their informed consent, pseudonymized clinical findings were reported by the referring geneticists/clinicians in a table shared by S.K and returned to him. Individual data are however not provided in the manuscript. Clinical data are presented in the form of aggregated data only.
Outcomes	n/a

Plants

Seed stocks	n/a
Novel plant genotypes	n/a
Authentication	n/a

Flow Cytometry

Plots

Confirm that:

☒ The axis labels state the marker and fluorochrome used (e.g. CD4-FITC).

☒ The axis scales are clearly visible. Include numbers along axes only for bottom left plot of group (a 'group' is an analysis of identical markers).

☒ All plots are contour plots with outliers or pseudocolor plots.

☒ A numerical value for number of cells or percentage (with statistics) is provided.

Methodology

Sample preparation	The PROTEOSTAT® Aggresome Red Detection Reagent was applied to cells that had been fixed with 4% paraformaldehyde and permeabilized according to the manufacturer's protocol.
Instrument	MACSQuant® Analyzer 10 Flow Cytometer (Milenyi Biotec)
Software	MACS Quantify Software (Miltenyi Biotec)
Cell population abundance	n/a, no cell sorting was performed.
Gating strategy	gating was used to remove cell debris and doublets/ clumped cells

☒ Tick this box to confirm that a figure exemplifying the gating strategy is provided in the Supplementary Information.
